# An Eye on Trafficking Genes: Identification of Four Eye Color Mutations in *Drosophila*

**DOI:** 10.1534/g3.116.032508

**Published:** 2016-08-23

**Authors:** Paaqua Grant, Tara Maga, Anna Loshakov, Rishi Singhal, Aminah Wali, Jennifer Nwankwo, Kaitlin Baron, Diana Johnson

**Affiliations:** * Department of Biological Sciences, The George Washington University, Washington, DC 20052; † Undergraduate Summer Research Program, Department of Biological Sciences, The George Washington University, Washington, DC 20052

**Keywords:** genetic analysis, vesicular transporters, LysM domain in eukaryotes

## Abstract

Genes that code for proteins involved in organelle biogenesis and intracellular trafficking produce products that are critical in normal cell function . Conserved orthologs of these are present in most or all eukaryotes, including *Drosophila melanogaster*. Some of these genes were originally identified as eye color mutants with decreases in both types of pigments found in the fly eye. These criteria were used for identification of such genes, four eye color mutations that are not annotated in the genome sequence: *chocolate*, *maroon*, *mahogany*, and *red Malpighian tubules* were molecularly mapped and their genome sequences have been evaluated. Mapping was performed using deletion analysis and complementation tests. *chocolate* is an allele of the *VhaAC39*-1 gene, which is an ortholog of the Vacuolar H^+^ ATPase AC39 subunit 1. *maroon* corresponds to the *Vps16A* gene and its product is part of the HOPS complex, which participates in transport and organelle fusion. *red Malpighian tubule* is the *CG12207* gene, which encodes a protein of unknown function that includes a LysM domain. *mahogany* is the *CG13646* gene, which is predicted to be an amino acid transporter. The strategy of identifying eye color genes based on perturbations in quantities of both types of eye color pigments has proven useful in identifying proteins involved in trafficking and biogenesis of lysosome-related organelles. Mutants of these genes can form the basis of valuable *in vivo* models to understand these processes.

*Drosophila melanogaster* is an extremely useful model organism for both classical genetics and molecular biology. It is also one of the earliest organisms to have its DNA sequence determined and extensively annotated. However, many of the genes identified and used in classical genetics have not been matched with their corresponding genes in the molecular sequence. This study identifies four genes coding for eye color mutations.

Certain eye color genes regulate vesicular transport in cells. Enzymes and other substances needed for pigment synthesis are transported to the pigment granule, a lysosome-related organelle (Dell’Angelica *et al.* 2000; Reaume *et al.* 1991; Summers *et al.* 1982). *D. melanogaster* has a red-brown eye color caused by the presence of two classes of pigments, pteridines (red) and ommochromes (brown). There are independent pathways for the synthesis of each (Reaume *et al.* 1991; Summers *et al.* 1982). Some eye color mutations are caused by the lack of individual enzymes in these pathways. Examples include bright red *vermillion* (tryptophan 2,3-dioxygenase), which is incapable of synthesis of any ommochromes, and dark brown *sepia* (GSTO4), which lacks an enzyme used to synthesize a subset of pteridines (Baillie and Chovnick 1971; Kim *et al.* 2006; Searles and Voelker 1986; Searles *et al.* 1990; Walters *et al.* 2009; Wiederrecht and Brown 1984). The first mutant described in *Drosophila*, *white*, lacks both types of pigments in the eye (Morgan 1910). The *white* gene codes for a subunit of an ABC transporter located in the membrane of the pigment granule, and is required for transport of substrates into pigment granules (Goldberg *et al.* 1982; Mackenzie *et al.* 1999; Summers *et al.* 1982). Lloyd *et al.* (1998) developed a hypothesis that single mutations causing perturbations in the amount of both types of pigments in the eyes could result from defects in genes associated with intracellular transport. They named these genes “the granule group.” Their concept has been supported by both identification of existing eye color genes for which the genomic coding sequences were originally unknown, and by studies in which induced mutations or reduced expression of orthologs of known transporter genes resulted in defective eye color phenotypes.

Vesicular trafficking is critical. Each cell in the body is highly organized with a number of compartments. These must contain specific proteins and have distinct characters, such as pH, as well as specific molecules attached to them. Vesicular trafficking is accomplished by a large number of protein complexes and individual effector proteins, and the list of these is still growing. One mode of delivery involves endocytic trafficking resulting in the sorting of proteins, lipids, and other materials and directing them to specific vesicles/organelles (Li *et al.* 2013). Many of the relevant proteins were first identified in yeast and have been highly conserved (Bonifacino 2014). *D. melanogaster* eye color genes provide *in vivo* metazoan models of these proteins and their interactions in trafficking. For example, the adaptor protein 3 (AP3) complex consists of four proteins: the β3 (RUBY), δ3 (GARNET), μ3 (CARMINE), and σ3 (ORANGE) subunits. It is involved in cargo selection for vesicles, transport of soluble *N*-ethylmaleimide-sensitive factor attachment protein receptor complexes, biogenesis of lysosomes, and lysosome-derived organelles, including formation of synaptic vesicles (Besteiro *et al.* 2008; Cowles *et al.* 1997; Faundez *et al.* 1998; Kent *et al.* 2012; Mullins *et al.* 1999, 2000; Ooi *et al.* 1997). The homotypic vacuole fusion and protein sorting (HOPS) complex in metazoans has four core proteins coded for by the *Vps16A*, *Vps11*, *Vps18* (*dor*), and *Vps33* (*car*) genes. Two additional proteins coded for by *Vps39* and *Vps41* (*lt*) can also be part of the complex. The HOPS complex participates in endocytic transport, endosome maturation, and fusion with lysosomes (Nickerson *et al.* 2009; Solanger and Spang 2013). Rab GTPases are molecular switches, active when coupled with GTP. The guanine exchange factor (GEF) catalyzes the conversion of GDP to GTP, activating its associated Rab GTPase. Rabs contribute to cargo selection, vesicle movement via microfilaments and actin, and fusion of membranes. Two eye color mutants, *lightoid* (Rab32) and *claret* (its putative GEF), have been shown to affect pigment granule morphology and autophagy; *lightoid*’s transcript has also been shown to be enriched in neurons. Human Rab32 participates in the transport of enzymes involved in melanin production to the melanosome, another lysosome-related organelle (Chan *et al.* 2011; Hutagalung and Novick 2011; Ma *et al.* 2004; Wang *et al.* 2012a; Zhen and Stenmark 2015). The human gene Lysosomal Trafficking Regulator Protein (LYST) is required for normal size and number of lysosomes and biogenesis of cytotoxic granules. Mutations in the gene are associated with Chediak–Higashi syndrome, which causes defects in immunity, prolonged bleeding, and oculocutaneous albinism. The fly ortholog, *mauve*, recapitulates some of these characteristics. It has overlarge pigment granules, abnormal eye color, and is susceptible to bacterial infection. It also lacks the ability to produce mature autophagosomes (Huizing *et al.* 2008; Rahman *et al.* 2012; Sepulveda *et al.* 2015). At least three sets of Biogenesis of Lysosome-related Organelle Complexes, BLOC1, BLOC2, and BLOC3, are required for normal development of these organelles (Dell’Angelica. 2004). The classic eye mutant, *pink*, is a member of BLOC2, an ortholog of Hermansky–Pudlak Syndrome 5 (HPS5) and shows genetic interactions with AP3 genes, *garnet* and *orange*, and the HOPS gene *carnation* (Di Pietro *et al.* 2004; Falcon-Perez *et al.* 2007; Syrzycka *et al.* 2007). Finally, *Drosophila* orthologs of genes originally identified as BLOC1 genes in other organisms have produced an abnormal eye color phenotype when their expression was inhibited by RNAi. These include four of the BLOC1 genes, *Blos1*, *Pallidin*, *Dysbindin*, and *Blos4* (Bonifacino 2004; Cheli *et al.* 2010; Dell’Angelica *et al.* 2000).

Using the perturbation in amounts of both ommochromes and pteridines as a criterion, we selected and identified the genomic sequence coding for each of four eye color genes in *D*. *melanogaster*: *chocolate* (*cho*), *maroon* (*ma*), *mahogany* (*mah*), and *red Malpighian tubules* (*red*). Two of the genes, *cho* and *ma*, are VATPase and *VPS16a* subunits, respectively. The roles of these annotated genes in vesicular transport have been previously characterized. The *mah* mutation also codes for a gene involved in transfer of amino acids to granules. Finally, the last gene, *red*, codes for a predicted protein with a LysM domain, but the protein’s function is unknown.

## Materials and Methods

### Nucleic acid isolation, RT-PCR, and production of transgenic flies

Fly DNA was isolated from single flies using the method of Gloor *et al.* (1993). The Parks *et al.* (2004) “5 Fly Extraction” was used to isolate purer DNA from groups of five to 10 flies at a time. The second technique was modified by using single microcentrifuge tubes and a tissue grinder for homogenizing. RNA was isolated using TRIzol (Life Technologies). cDNA was prepared using Superscript II (Life Technologies) and an oligo dT primer. Genomic DNA for wild-type versions of *cho*, *ma*, and *mah* and cDNA from the *CG12207* transcript A were amplified by PCR and TOPO-TA cloned (Life Technologies). Each gene’s DNA/cDNA was subcloned into the pUAST vector (*Drosophila* Genomic Resource Center) in order to use the GAL4/UAS technique (Brand and Perrimon 1993; Duffy 2002). Clones were sequenced to verify that they had the wild-type sequence. The constructs were isolated with Plasmid Midi Prep kits (Qiagen). Injections of the pUAST clones to produce transgenic flies were performed by Rainbow Transgenic Flies.

### Fly stocks and crosses

Fly stocks were maintained at 25° on Instant *Drosophila* Medium (Carolina Biological). Jim Kennison provided the EMS-induced *red^K1^* stock and its OreR progenitor. Other stocks were obtained from the Bloomington and Exelixis Stock Collections (Table 1). Deletions were made using the Flp-FRT methods described by Parks (Parks *et al.* 2004). The chromosome sequence coordinates of specific existing deletions and deletions that were made are shown in Supplemental Material, Table S1. Recombinants were chosen based on their eye color. PCR was used to verify the deletions.

**Table 1:** Stocks used for locating eye color mutants

*cho* Mapping	All Autosomal Mapping	*mahogany* Mapping, cont’d.
P{XP}*XPG-L* Exelixis Stock Collection	P{*ry*^+t7.2^*=hsFLP}1*, *y^1^ w^1118^*; Dr^Mio^/TM3 ry* Sb^1^	*w^1118^;Df(3R)P{XP}CG31121^d06890^* to *PBac{WH}^f01730^/TM6B*, *Tb* Constructed by authors
*w^1118^/Binsinscy*	*w^1118^*; *wg ^Sp-1^*/*CyO*; *sens^Ly-1^/TM6B*, *Tb^1^*	
*w^1118^*; *MKRS*, *P*{*ry^+t7.2^* = hsFLP}86E/TM6B, *Tb^1^*		*red* Mapping
*w^1118^*/*FM7c*	*maroon* Mapping	*w^1118^*; *Df(3R)Exel7321/TM6B*, *Tb^1^*
*y^2^ cho^2^ flw^1^*	*ma^1^ fl^1^*	*w^1118^*; *Df(3R)Exel6267*, *P{w^+mC^ = XP-U}Exel6267/TM6B*, *Tb^1^*
*Df(1)10-70d*, *cho^1^ sn^3^*/*FM6*	*w^1118^*; *Df(3R)BSC507/TM6C*, *Sb^1^ cu^1^*	*w^1118^*; *PBac{ w^+mC^ = RB}su(Hw)e^04061^/TM6B*, *Tb^1^*
*Df(1)ED6716 w^1118^/FM7h*	*w^1118^*; *PBac{RB}Aats-trp^e00999^/TM6B* Exelixis Stock Collection	*w^1118^*; *P{XP}trx^d08983^/TM6B*, *Tb^1^*
*w^1118^ P+PBac{XP3.WH3}BSC877/FM7h/Dp(2;Y)G P{hs-hid}Y*	w^1118^; P{XP}^d00816^/TM6B Exelixis Stock Collection	*w^1118^*; *Df(3R)P{XP}trx^d08983^* to *PBac{RB}su(Hw)^e04061^/TM6B*, *Tb^1^* Constructed by authors
*Df(1)BSC834 w^1118^/Binsinscy*	*w^1118^*; *Df(3R)Exel9036*, *PBac{WH}Exel9036/TM6B*, *Tb^1^*	
*w^1118^*; *P{XP}^d03180^* Exelixis Stock Collection	*w^1118^;Df(3R)ED5339*, *P{3′.RS5+3.3′}ED5339/TM6C*, *cu^1^ Sb^1^*	
*w^1118^*; *PBac{RB}VhaAC39-1^e04316^* Exelixis Stock Collection	*w^1118^;Df(3R) PBac{RB}Aats-TrpRS^e00999^* to *P{XP}^d00816^/TM6B*, *Tb^1^* Constructed by authors	Additional Stocks for Rescue Crosses
*w^1118^*; *P{^w+mC^ = XP}yin^d02176^*		w*; *P{w^+mc^ GAL4-ninaE.GMR}12*
*P{XP}ec^d00965^* Exelixis Stock Collection	*mahogany* Mapping	w*;Cy/P{w^+mc^ GAL4-ninaE.GMR}12 Constructed by authors
*PBac{WH}^f06086^* Exelixis Stock Collection	*mah^1^*	w*; *Kr^If-1^/CyO*
*w^1118^*;*Df(1) P{XP}ec^d00965^* Exelixis Stock Collection	w^1118^; PBac{WH}^f01730^/TM6B Exelixis Stock Collection	w*; *Kr^If-1^/CyO*; *Df(3L)Ly*, *sens^Ly-1^/TM6C*, *Sb^1^ Tb^1^*
*PBac{WH}^f06086^* Exelixis Stock Collection	*w^1118^*; *P{w^+mc^ = XP}CG31121^d06890^/TM6B*	*w^1118^*; *Kr^If-1^/CyO*; *TM3*, *Sb^1^/D^1^*
*w^1118^*;*Df(1) P{XP}ec^d00965^* to *PBac{WH}^f06086^/FM7h* Constructed by authors	*w^1118^*; *Df(3R)BSC494/TM6C*, *Sb^1^ cu^1^*	w*; *Kr^If-1^/CyO*; *CxD/TM6C*, *Sb^1^ Tb^1^*
*w^1118^ PBac{RB}VhaAC39-1^e04316^* to *P{XP}yin^d02176^/FM7h* Constructed by authors	*w^1118^*; *Df(3R)Exel6200*, *P{XP-U}Exel6200/TM6B*, *Tb^1^*	w*; *Kr^If-1^/CyO*; *TM3*, *Ser^1^/D^1^*
*w^1118^*; *Df(1)P{XP}^d03180^* to *PBac{RB}VhaAC39-1^e04316^/FM7h* Constructed by authors	*w^1118^*; *Df(3R)BSC318/TM6C*, *Sb^1^ cu^1^*	*w^1118^*; *TM3*, *Sb^1^/CxD*

Unless otherwise noted, the stocks were obtained from the Bloomington *Drosophila* Stock Center.

Deletion mapping crosses were made between each deletion stock (Table 2) and its corresponding homozygous mutant stock. Rescue crosses required that eye color phenotypes be evaluated in *w^+^* flies. The GAL4/UAS system was used (Brand and Perrimon 1993; Duffy 2002). The driver employed for eye specific expression was Gal4-ninaE.GMR12. Stocks with balancers were used to produce *w^+^*; *Cy/P{GAL4-ninaE.GMR}12* homozygous mutant stocks for *ma*, *mah*, and *red*. These flies were crossed at 27° with the *Cy*/pUAST transgene stocks that were also homozygous for the appropriate third chromosome mutant allele and the phenotype was assayed in the F_1_s. For the X-linked *cho* gene, *w^+^cho/w^+^cho*; *Cy*/*P{GAL4-ninaE.GMR}12* females were crossed with *w*Y; *Cy/pUAST-cho* transgene stock flies and the phenotype was evaluated in the male F_1_. Rescue was assayed for at least three independent transgene lines for each gene.

**Table 2:** Existing deletions or transposable elements used to produce deletions

Gene Deletion or Transposable Elements	Complements Mutant
*chocolate*	
*P{XP}*^*d03180*^ and *PBac{RB}VhaAC39-1*^*e04316*^	Yes
*PBac{RB}VhaAC39-1*^*e04316*^ and *P{XP}yin*^*d02176*^	Yes
*P{XP}ec*^*d00965*^ and *PBac{WH}f06086*	No
*Df(1)BSC834*	Yes
*Df(1)BSC877*	Yes
*Df(1)ED6716*	No
*maroon*	
*Df(3R)ED5339*	No
*Df(3R)Exel9036*	Yes
*Df(3R)BSC507*	No
*mahogany*	
*Df(3R)BSC318*	No
*Df(3R)Exel6200*	Yes
*Df(3R)BSC494*	Yes
*P{XP}CG31121*^*d06890*^ and *PBac{WH}f01730*	Yes
*red* *Malpighian tubules*	
*Df(3R)Exel6267*	No
*Df(3R)Exel7321*	Yes
*P{XP}trx*^*d08983*^ and *PBac{RB}su(Hw)*^*e04061*^	No

Data from complementation experiments are given for each deletion.

### Photomicroscopy

Flies were photographed using a Leica DFC425 digital camera mounted on a Leica M205A stereomicroscope. A series of 15–70 images were taken at different focal planes with the software package Leica Application Suite version 3.0 (Leica Microsystems, Switzerland) and assembled using the software Helicon Focus 6 (Helicon Soft, Ukraine).

### Sequence analysis

Sequencing was performed by the DNA Analysis Facility on Science Hill at Yale University, the Biological Sciences Sequencing Facility at the George Washington University, and Macrogen USA. Primers for cloning and sequencing are listed in Table S2. Sequences were compiled using Sequencher (Gene Codes). Predicted protein alignments were performed using T-Coffee or PSI-Coffee (Di Tommaso *et al.* 2011; Notredame *et al.* 2000) or PRALINE. PSI-Coffee alignment figures were produced using Boxshade (Source Forge) and PRALINE (Simossis and Heringa 2003; Simossis *et al.* 2005).

### Data availability

Sequences of mutant alleles have been deposited in Genbank (accession nos. KU665627 – *cho^1^*, KU682283 – *ma*^1^, KU682282 – *mah*^1^, *red* KU711835 - *red*^1^ and *red* KU711836 - *red*^K1^). Other gene sequences are available by request. Aligned nucleotide sequences for the *red* mutants and OreR are in Figure S1. Table S2 lists the primers used in cloning and sequencing. Nucleotide changes in *cho*, *ma*, and *mah* are in Table S3. The identifiers for all proteins used in alignments are given in Table S4. The distribution of nucleotide substitutions in the two *red* stocks and OreR are shown in Table S5. 

## Results

### cho codes for a subunit of vesicular ATPase

*cho* is an X-linked recessive eye color mutation that is also associated with brown pigmentation in the Malpighian tubules. It was originally described by Sturtevant (Lindsley and Zimm 1968; Sturtevant 1955). Both the amounts of ommochromes and pteridines present in *cho* eyes are decreased compared to wild type (Ferre *et al.* 1986; Reaume *et al.* 1991). The cytological position of *cho* is 3F1–3F4 and Sturtevant placed it close to *echinus* (*ec*). Three overlapping deficiencies, Df(1)BSC834, Df(1)ED6716, and Df(1)BSC877, which together removed regions around and including *ec*, were used for the first deletion mapping (Table 2). Only heterozygotes for *cho* and Df(1)ED6716 showed the cho phenotype, indicating that *cho* was in the region removed exclusively by Df(1)ED 6716 (Table 2). Genomic deficiencies were made using the Flp-FRT method (Parks *et al.* 2004). Deletion mapping with these identified a 47.8-Kbp region that contained the *cho* gene. Within it were three candidate genes: *VhaAC39-1*, a subunit of vacuolar ATPase; *CG42541*, a member of the Ras GTPase family; and *CG15239*, which has an unknown function (Table 2).

**Figure 1:**
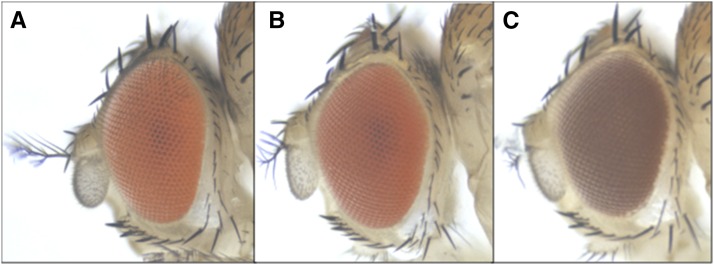
The *VhaAC39-1* gene complements the *chocolate* gene. (A) Wild-type genotype and phenotype. (B) *cho*/Y with transgene showing the wild-type phenotype. The transgenic male is hemizygous for the mutant allele and carries the Gal4 driver from w*; *P{GAL4-ninaE.GMR}12* and one copy of the VhaAC39-1 transgene. (C) *cho*/Y showing the mutant phenotype.

**Figure 2:**
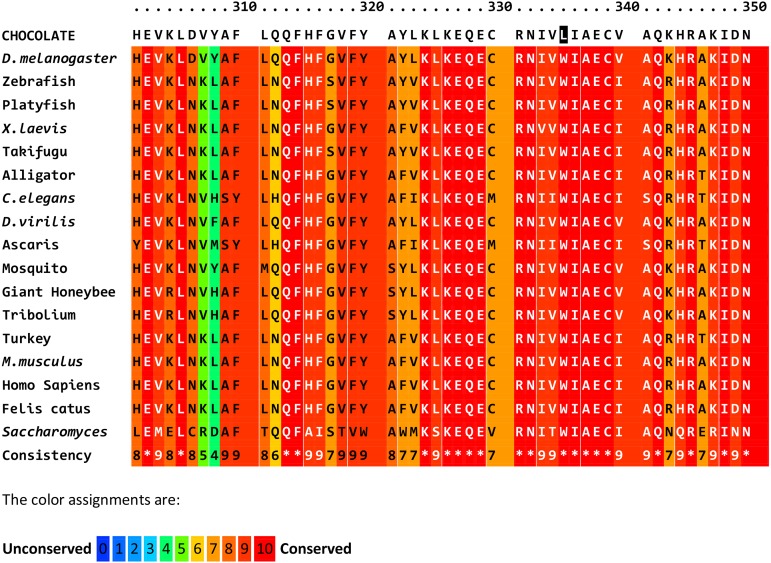
Alignment of predicted partial protein sequences for CHOCOLATE orthologs in insects, fish, reptiles, birds, mammals, and yeast. Multiple sequence alignment, conservation scoring, and coloring were performed by PRALINE. 0 is the least conserved alignment position, increasing to 10 for the most conserved alignment position. Asterisks in the consistency sequence indicate identity in all sequences. The CHO sequence is not included in the consistency rating. The predicted CHO sequence for the region surrounding the missense mutation (shaded) is in the first line. The orthologous sequences were obtained for some vertebrates and yeast. The whole protein is highly conserved and the tryptophan at position 335 is constant except for the CHO sequence which has leucine. Species, gene, and protein identifiers are in Table S4.

Each of these genes was amplified from *cho* DNA and sequenced. The coding regions of *CG15239* and *CG42541* did not contain any nonsynonymous sequence changes in the *cho* mutant flies. The coding region of *VhaAC39-1* had a change from guanine to thymine at position X:3,882,405 that resulted in a nonsynonymous change, W330L, in the deduced protein sequence (Table 3 and Table S3). The UAS-VhaAC39-1 transgene (carried on chromosome 2) and the Gal4-ninaE.GMR12 driver were used to fully rescue *cho/cho* stocks (Figure 1). The residue at position 330 is highly conserved. Alignments of predicted orthologs from insects, other invertebrates, including yeast and vertebrates all showed a tryptophan at their corresponding positions (Figure 2). The conservation and phenotype change caused by *cho* mutant alleles both show the importance of this tryptophan in protein function.

**Table 3:** Nonsynonymous differences, deletions, and insertions between the *Drosophila melanogaster* genome sequence and mutant alleles of *maroon*, *chocolate*, *mahogany*, and *red Malpighian tubules*

Position	DNA Change	Nonsynonymous Changes
X	*VhaAC39-1-chocolate*	
X:3,882,405	G > T	W330L
3R	*Vps16A-maroon*	
9267133	G > A	M4I
9267260	G > A	A24T
9268607–9268615	Deletion	Deletion 422-I M R-424
9269662	A > T	E712D
3R	*CG13646-mahogany*	
24949138	*roo* insert	
24949334	T > C	I460T*^a^*
3R	*CG12207-red*	
	*red^1^*	
14300442	A > C	N67H*^b^*
	*red^K1^*	
14298973	G > A	G51S*^b^*

a This part of the exon may not be translated when the *roo* LTR is present.

b The amino acid positions listed are for isoforms PA, PD, and PE. In isoforms PB, PF, and PG, the positions are N90H for *red^1^* and G74S for *red^K1^*.

### ma codes for a component of the HOPS complex, *Vps16A*

The *ma* allele is recessive and homozygotes have darker eyes than normal and yellow Malpighian tubules (Bridges 1918). Homozygotes also have decreased amounts of both pteridines and ommochromes (Nolte 1955). Deletion mapping results (Table 2) indicated that the region in which *ma* resides contains *Vps16A*, a gene involved in endosomal transport and eye pigment (Pulipparacharuvil *et al.* 2005); *Aats-trp*, a tRNA synthase (Seshaiah and Andrew 1999); *CG8861*, a predicted Lymphocyte Antigen super family 6 gene (Hijazi *et al.* 2009); *HP1E*, a member of the heterochromatin protein 1 family (Vermaak *et al.* 2005); and *CG45050*, a gene coding for a protein with Zinc finger domains (dos Santos *et al.* 2015).

**Figure 3:**
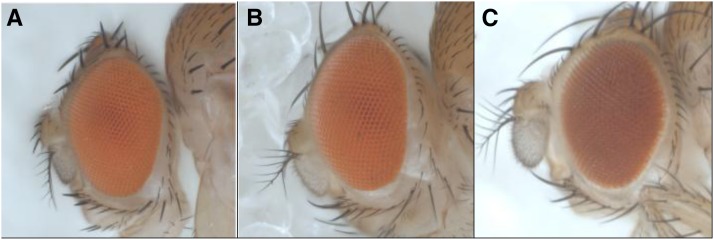
The *Vps16A* wild-type allele complements the *maroon* mutation. (A) Wild-type genotype and phenotype. (B) *ma/ma* mutant genotype plus the *Vps16A* transgene and *P{GAL4-ninaE.GMR}12* driver produces a wild-type phenotype. (C) *ma/ma* mutant genotype and phenotype.

The *Vps16A* gene was sequenced in *ma* flies because of its role in the HOPS complex. The DNA sequence has 10 nucleotide substitutions and a 9-bp deletion (Table S3). The protein has three missense substitutions and a deletion of three contiguous amino acids (Table 3). Homozygous mutant *ma^1^* flies had a near wild-type phenotype when rescued by the UAS-*Vps16A* transgene driven by the Gal4-ninaE.GMR12 driver (Figure 3).

**Figure 4:**
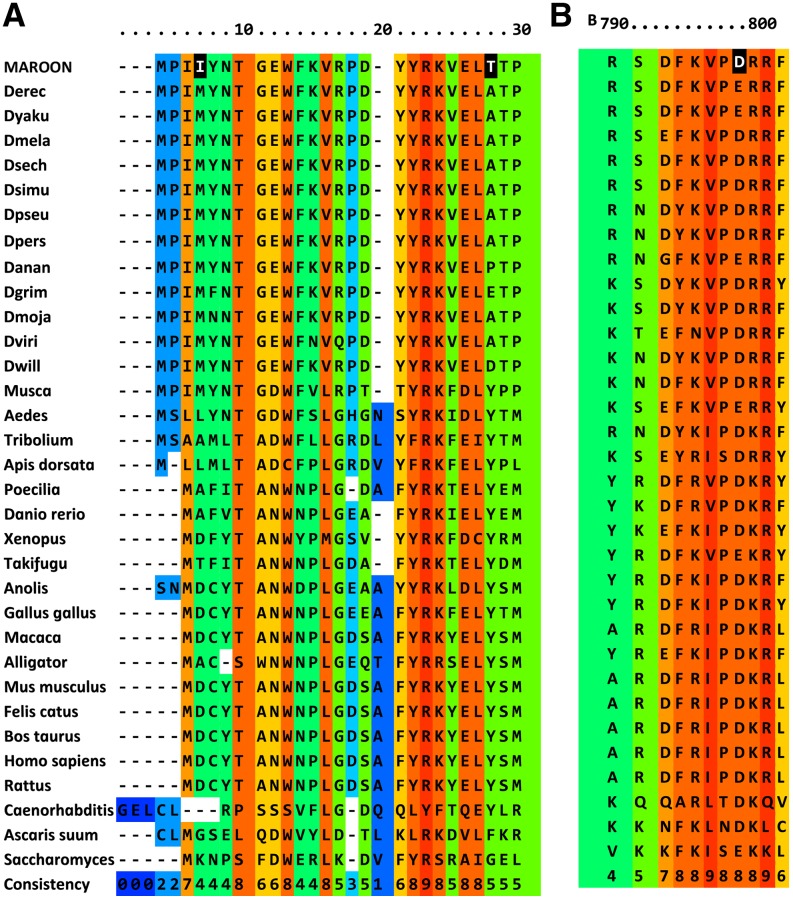

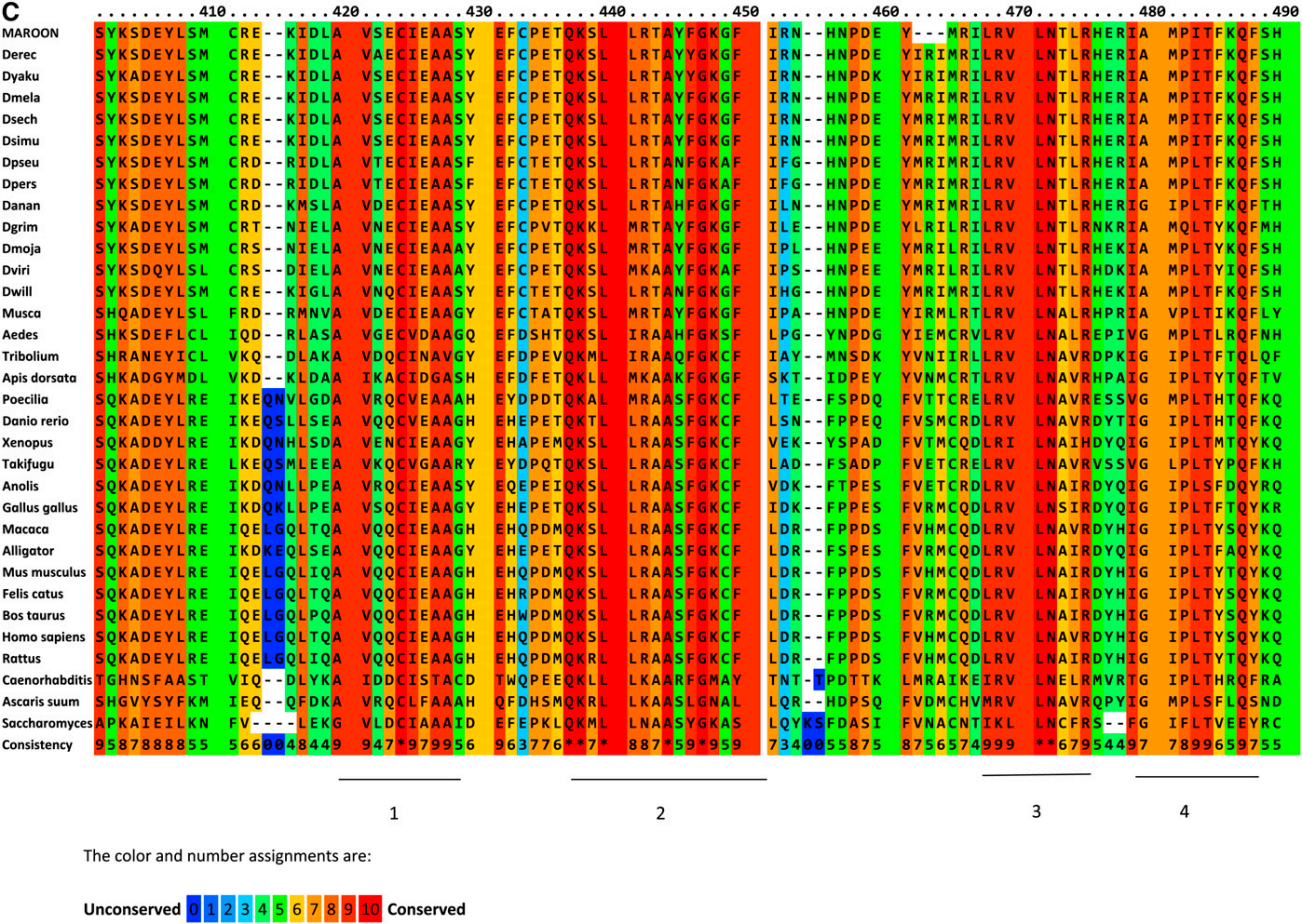
A PRALINE alignment of regions of the predicted MAROON protein which have changed compared to the *D. melanogaster* sequence. Alignment, conservation scoring and coloring was performed by PRALINE. The *ma* sequence is not included in the consistency rating. 0 is the least conserved alignment position, increasing to 10 for the most conserved alignment position. Asterisks in the consistency line indicate identity for all sequences. Species, gene and protein identifiers are in Table S4. MA amino acid changes are shaded black in the first line for each alignment. (A) The first two amino acid changes are in regions with low conservation. Note, however, that the residue at position 7 is not found in any other species. The M at that site is conserved in all *Drosophila*. The A to T change at position 28 is also not found in other species. The position is variable in insects, but vertebrates usually have a Y. (B) The E to D change at position 797 is in a moderately conserved area. D is found in many organisms at this site. (C) The deletion of three amino acids in the MA protein shown in line one at positions 461–463 lies between two sets of conserved sequences, 1, 2, 3, and 4 (underlined). The distance between the 2 and 3 regions is conserved in all species shown except *C. elegans* and *S. cerevisiae*.

The cause of impaired protein function is unclear. The first two substitutions (M4I and A24T) are not conservative and are found in variable regions in the protein (Figure 4A). The aspartic acid of the E712D substitution is found in the corresponding site in the majority of *Drosophila* species and in many other species (Figure 4B). The deletion sequence is in a region with moderately conservative changes between amino acids and it does lie between two regions that are conserved from fly to man (Figure 4C). The spacing between these conserved regions is invariant among the investigated species, with the exception of *Caenorhabditis elegans* and *Saccharomyces cerevisiae*.

### mah contains domains found in amino acid transporters and permeases

*mah* is a recessive eye color mutant on the right arm of the third chromosome with a recombination map position of 88. It was discovered by Beadle (Lindsley and Zimm 1968). Homozygotes have decreases in ommochrome pigments and six of 10 pteridine pigments (Ferre *et al.* 1986).

Deficiency mapping showed that the possible *mah* genes were *Nmnat* and *CG13646* in the 96B cytological region (Table 2). *Nmnat* is Nicotinamide mononucleotide adenylyltransferase and is involved in NAD synthesis and photoreceptor cell maintenance (Zhai *et al.* 2006). The *CG13646* gene contains transmembrane domains and an amino acid transporter domain (dos Santos *et al.* 2015; Romero-Calderon *et al.* 2007). Along with functions that could relate to pigmentation, both genes had expression patterns consistent with an eye color gene (dos Santos *et al.* 2015) *Nmnat* and *CG13646* were sequenced using DNA from the *mah* strain.

**Figure 5:**
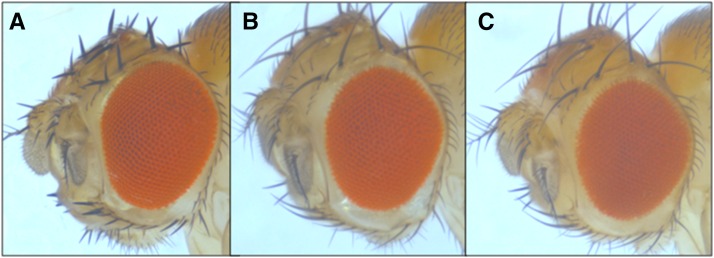
The *CG13646* gene rescues the *mahogany* gene. (A) Wild-type genotype and phenotype. (B) *mah/mah* mutant genotype and *CG13646* transgene under the control of the Gal4 driver *P{GAL4-ninaE.GMR}12* shows the wild-type phenotype. (C) *mah/mah* genotype shows the mutant phenotype.

The *Nmnat* allele sequence matched the Flybase sequence in its coding region and introns. The *CG13646* gene had 10 single base pair substitutions and an insertion of 433 bp, including a duplication of a 5-bp insert sequence in its fifth exon (Table S3). The additional DNA is a solo insert of a *roo* LTR (Meyerowitz and Hogness 1982; Scherer *et al.* 1982). For *CG13646*, only one substitution mutation was nonsynonymous, a T > C at 3R:24949334 causing an I460T change if that position were transcribed and translated (Table 3). The UAS-*CG13646* transgene under the control of the eye-specific GAL4-ninaE.GMR12 driver rescued *mah/mah* flies (Figure 5).

**Figure 6:**
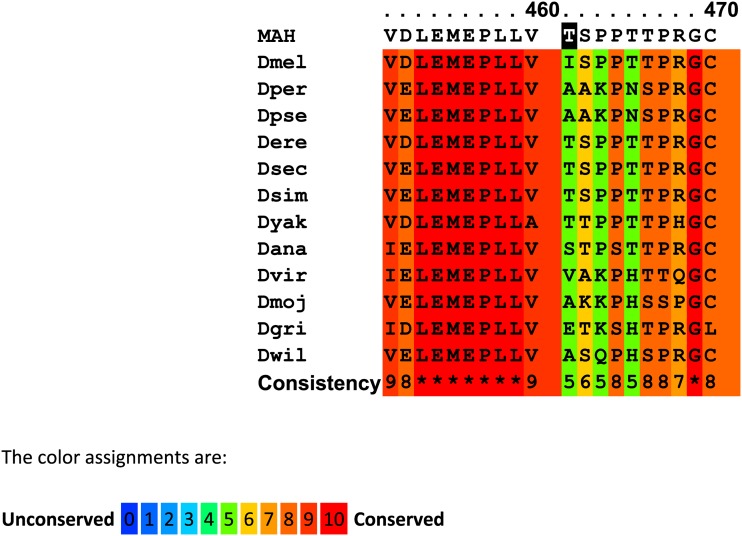
PRALINE alignment and consistency scores of predicted residues 450–470 with the MAHOGANY protein sequence in *Drosophila* species. 0 is the least conserved alignment position, increasing to 10 for the most conserved alignment position. Asterisks in the consistency rating indicate identity for all. The MAH sequence is not included in the consistency rating. The change in sequence in the MAH protein is shaded black in the first line. A number of other species, *D. erecta*, *D. simulans*, *D. yakuba*, and *D. sechellia*, have a T at the same position as mahogany. Species, gene, and protein identifiers are in Table S4.

The insertion would be expected to cause production of a truncated inactive protein product. If the translation of the last exon proceeds through the insert, there will be an early termination signal adding three amino acids coded for by *roo* and deleting 130 amino acids. Comparing the *CG13646* orthologous proteins in 12 *Drosophila* species shows the threonine substitution is at the corresponding position in *D. melanogaster’s* close relatives, *D. simulans*, *D. erecta*, and *D. sechellia*, implying that this substitution would be functional if the *mah* transcript were properly spliced and translated (Figure 6).

Unlike the genes discussed above, *mah*’s protein product shows very high sequence similarity to predicted orthologous proteins in other *Drosophila* species but markedly decreased similarity to orthologs in other insects (Figure S1A). It shows relatively low similarity to vertebrate proteins. While Psi Blast searches with the *CG13646* protein found sequence similarity with human and mouse GABA vesicular transporters, another *D. melanogaster* predicted protein, VGAT, shows greater sequence similarity and is the presumptive ortholog of the mammalian protein (Figure S1B).

### red is coded for by a gene with a LysM domain and an unknown function

The *red* gene’s phenotype is caused by a recessive allele producing flies with dark red-brown eyes and rusty red-colored Malpighian tubules. This gene is located on the third chromosome at cytogenetic position 88B1-B2 and was discovered by Muller (Lindsley and Zimm, 1968). *red* flies show decreases in both ommochromes and some pteridines (dos Santos *et al.* 2015; Ferre *et al.* 1986).

Deletion mapping revealed *red* was coded for by one of three genes, *CG12207*, CG3259, or *su(Hw)* (Table 2). The *su(Hw)* gene codes for a DNA-binding protein and mutants have well described phenotypes that do not involve the eyes. The CG3259 gene is involved in microtubule binding and the *CG12207* gene codes for a product of unknown function. The exons of *CG12207* and the complete CG3259 gene were sequenced in three stocks, *red^1^*, *red^K1^*, and the OreR stock that is the *red^K1^* progenitor.

Compared to the Genbank reference sequence for *CG12207* and *CG3259*, the three stocks had substitutions at 34 sites for *CG12207*. The most telling comparison is *red^K1^* *vs.* its progenitor. The *red^K1^* stock contained four changes that were absent from OreR (Table 4 and Table S5). One was a missense change producing G51S in the LysM domain of protein isoform A (Table 3). The others were two synonymous changes and one substitution in an untranslated region. The *red^1^* stock had 26 substitutions compared to the Genbank reference sequence and 12 compared to OreR (Table S5). It carried only one missense mutation, A to C, that produced an N67H change in the LysM domain of the protein isoform A (Table 3 and Table 4).

**Table 4:** Types and numbers of substitutions in *red^1^*, *red^K1^*, and OreR stocks for *CG12207* and CG3259 compared with the Genbank reference sequences for *CG12207* and *CG3259*

Gene	Type	Stock		
		*red^1^*	*red^K1^*	OreR
*CG12207*	Missense	1	1	0
*CG12207*	Synonymous	4	2	0
*CG12207*	UTR	21	15	14
Total		26	18	14
*CG3259*	Missense	9	5	5
*CG3259*	Synonymous	9	6	4
*CG3259*	UTR	2	3	3
Total		20	14	12

The *CG3259* sequences from the three stocks had 25 substitutions compared to the Genbank reference sequence (Table 4). The *red^K1^* stock had two changes in sequence that were missing in OreR, one synonymous and one in an untranslated region. These two stocks shared the same predicted protein sequence with four amino acids that varied from the Genbank reference protein sequence. The *red^1^* flies shared the replacements coding for the four amino acid substitutions and had four more missense changes, resulting in a substitution of eight amino acids compared with the Genbank reference sequence (Table 4 and Table S5).

The nucleotide sequences for both genes from OreR and the two *red* stocks were compared to the *Drosophila* Genomic Reference Panel, a database showing nucleotide polymorphisms in a panel of 200 inbred lines of *D. melanogaster* (Mackay *et al.* 2012). All of the *CG12207* replacements were found in the panel, except for each missense change in *red^1^* and *red^K1^* (Figure S2). All of the CG3259 changes also were found in the panel, except one shared by OreR and *red^K1^*. That substitution resided in an intron. When the CG3259 predicted protein sequences were compared in 11 *Drosophila* species, most amino acid changes were found in other species and were at variable sites in the protein sequence (data not shown).

**Figure 7:**
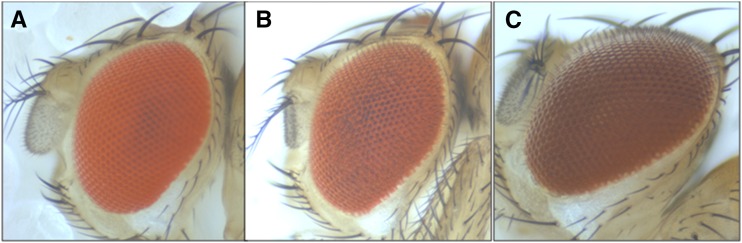
CG 12207 partially complements the *red/red* genotype. (A) Wild-type genotype and phenotype. (B) *red/red* genotype and partially wild-type phenotype with a *CG12207* transgene and the *P{GAL4-ninaE.GMR}12* driver present. (C) Red mutant phenotype in *red/red* fly.

Given the presence of unique amino acid changes in *red* *CG12207* sequences, the UAS-*CG12207* cDNA-A construct was chosen for a rescue experiment. It was able to partially or fully complement *red* flies when expressed with the *GAL4-ninaE.GMR12* driver, demonstrating that *CG12207* is the *red* gene (Figure 7). These data are consistent with the Johnson Laboratory analysis comparing the reference gene sequence to an earlier restriction map that included the *red* gene (Breen and Harte 1991) and the predictions of Cook and Cook found in Flybase (FBrf0225865).

**Figure 8:**
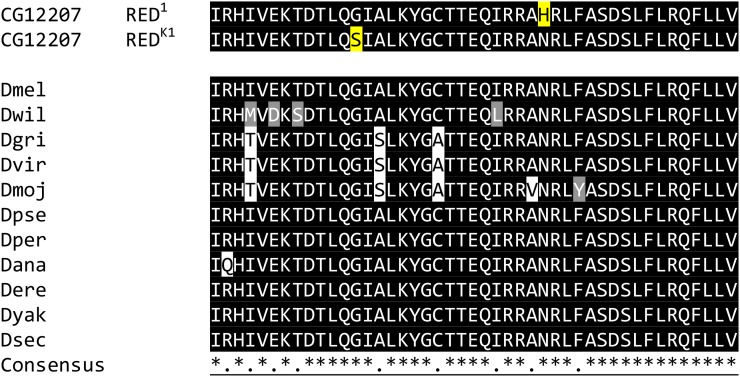
Alignment of LysM Domains in *CG12207* orthologs in 11 *Drosophila* species and in RED^1^ and RED^K1^. The multiple sequence alignment was performed by PSI-Coffee and the illustration made using BoxShade. RED^1^ and RED^K1^ proteins were not included in the consensus calculation. Residues that match the consensus sequence are shaded black. Gray regions represent changes to similar amino acids. White regions indicate substitutions to less similar amino acids. Asterisks in the consensus denote identity in all sequences. The *red^1^* allele (top line) codes for a substitution of G to S and *red^K1^* (second line) produces an N to H substitution (both marked in yellow). In wild-type *D. melanogaster* and the other 10 species, these sites are conserved. Species, gene, and protein identifiers are in Table S4.

The single domain identified in *CG12207* is the LysM domain and it is the site of both *red^1^* and *red^K1^* missense mutations. It is present in all six transcripts of the gene, but the function(s) of the predicted proteins are unknown. The domain in *CG12207* is highly conserved in predicted *Drosophila* orthologs (Figure 8). Further, the LysM domain is present in Eubacteria, plants, animals, and fungi.

**Figure 9:**
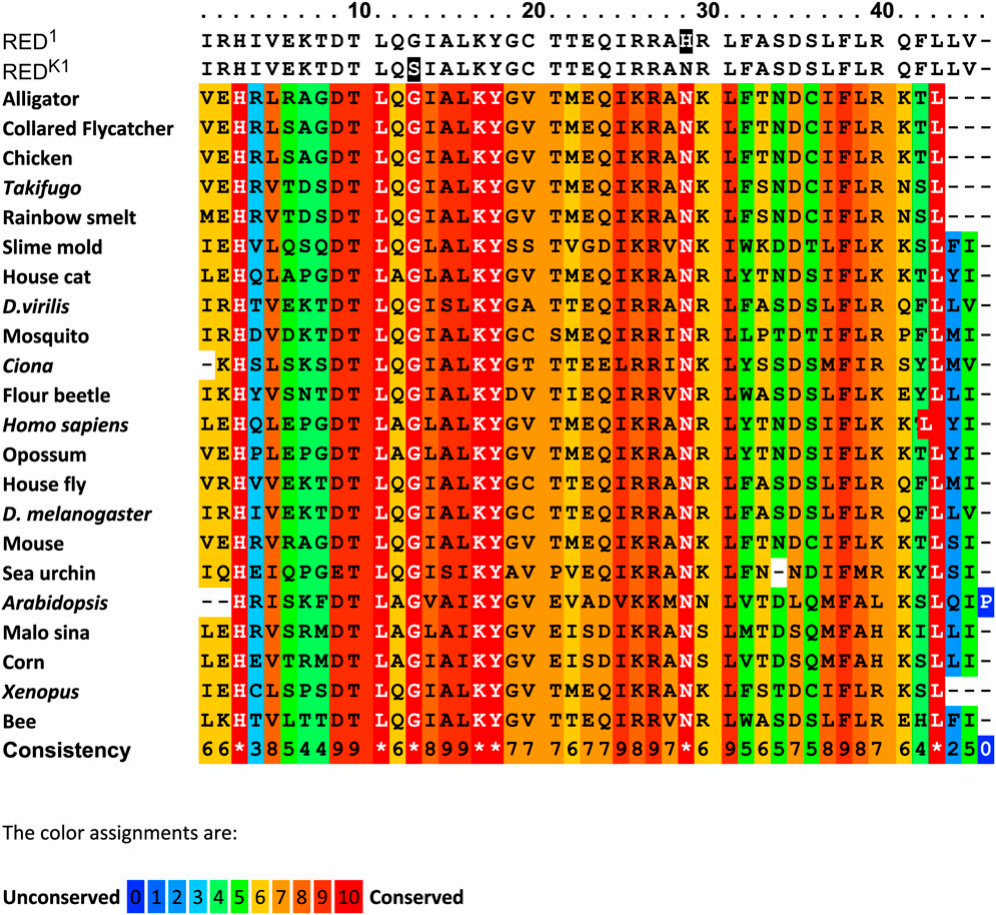
PRALINE alignment of predicted LysM domains from a variety of animals and plants. Asterisks in the consistency line indicate identity for all sequences. The RED^1^and RED^K1^ protein sequences are not included in the consistency rating. Sequences were chosen by their similarity to the CG12207 LysM domain sequence. The sites that were mutated in RED^*1*^ and RED^*K1*^ (shaded black) are normally completely conserved from rice and corn to man. All species, protein, and gene identifiers are in Table S4.

Examples of LysM domains similar in sequence to that found in *red* were identified b querying NCBI Blast using the *CG12207* LysM domain. These were aligned using T-Coffee (Notredame *et al.* 2000) and the G and N sites mutated in *red* alleles were perfectly conserved from rice to man (Figure 9). In addition, the N site appears conserved in other LysM motifs (Laroche *et al.* 2013; Zhang *et al.* 2009). Glycine is often found in tight turns in proteins and even a seemingly neutral change to serine may influence protein conformation (Betts and Russell 2003). While changes of asparagines to histidines are found in proteins, the exclusive presence of asparagines in the LysM domains examined supports the idea that the amino acid is important for normal protein function.

## Discussion

### The cho gene is essential and functions to acidify cellular compartments

The *cho* gene, *VhaAC39-1*, codes for Vacuolar H^+^ ATPase AC39 subunit *d*. The V-ATPase is found in all eukaryotes and is present in lysosomes, endosomes, and clathrin-coated vesicles. The two sectors of V-ATPases are *V*_1_ and *V*_0_, each of which contains multiple proteins. *V*_1_ is found outside the organelle/vesicle and interacts with ATP, ADP, and phosphate. *V*_0_ is found in the plasma membrane and transports H^+^ into the compartment (Beyenbach and Wieczorek 2006; Marshansky *et al.* 2014). Subunit *d* of the *V*_0_ complex may function in connecting the *V*_1_ complex to the *V*_0_ complex through interactions with *V*_1_ protein subunits (Allan *et al.* 2005). Intracellular V-ATPases play important roles in trafficking, such as separation of ligands and receptors in endosomes, and degradation in lysosomes (Wang *et al.* 2012b). Acidification regulates and mediates the trafficking of cellular receptors and ligands, such as Notch and Wnt.

The mutation in the *cho* gene is a tryptophan-to-leucine mutation. Tryptophan is the rarest amino acid in eukaryotic proteins, while leucine is the most common (Gaur 2014). Owegi *et al.* (2006) investigated the effects of a change of the corresponding tryptophan to alanine (W325A) in yeast. Loss of the tryptophan decreased the assembly of the *V*_0_*V*_1_ active enzymes, ATPase activity, and proton transport to about 10% of the normal rate.

The *cho/VhaAC39-1* gene has several other characteristics that are consistent with its role as an important component of trafficking. RNAi experiments demonstrated that its knockdown in *D. melanogaster* resulted in complete lethality (Mummery-Widmer *et al.* 2009). Its ortholog in mice is also essential (Miura *et al.* 2003). The viability of *cho* flies indicates a malfunction of VHAAC39-1 that results in a decrease in activity *vs.* a loss of protein, as seen in knockdowns. Further, *cho* is ubiquitously expressed (Chintapalli *et al.* 2007; Hammonds *et al.* 2013; Tomancak *et al.* 2002, 2007). Studies of *cho*’s effects in all parts of the body could reveal gene interactions between VHAAC39-1 and proteins of other mutant genes participating in the same pathways.

### The ma gene is essential and participates in the HOPS complex used in trafficking, endosome maturation, and fusion with lysosomes

The *ma*/*Vps16A* gene product is one of four core proteins in the HOPS complex in metazoans. This role has been confirmed in flies. Pulipparacharuvil *et al.* (2005) have shown that *VPS16A* complexes with DOR and CAR proteins. Takáts *et al.* (2014) demonstrated that the HOPS complex is required for fusion of autophagosomes with the lysosomes. Disruption of the HOPS complex also resulted in increased metastasis and growth of tumors in *Drosophila* (Chi *et al.* 2010). Recent work in humans has shown that VPS 16 is required to recruit VPS 33A to the HOPS complex. The lack of either of these proteins prevented the fusion of lysosomes with endosomes or autophagosomes (Wartosch *et al.* 2015).

In *D. melanogaster*, a *VPS16A* knockdown in the eye resulted in a change in eye color and retinal degeneration due to defects in lysosomal delivery and the formation of pigment granules. An organism-wide knockdown of *VPS16A* caused death (Pulipparacharuvil *et al.* 2005). Like *cho*, *ma* is an essential gene and the *ma* allele produces a partially functional protein. In addition, *ma* RNA is maternally deposited and the gene is expressed widely in larvae and adults (Chintapalli *et al.* 2007; Hammonds *et al.* 2013; Tomancak *et al.* 2002, 2007). The study of *ma* mutant flies for other possible interacting genes would produce an *in vivo* system capable of revealing new gene networks and their sites of action.

### The red gene’s product has an unknown function and a LysM domain, which is part of a superfamily found in bacteria, plants, and animals

The role of the *red*/*CG12207* gene product is unknown. The fact that the two *red* mutant alleles had missense substitutions in their LysM domains indicates that the domain is important for the protein’s function. In animals, the LysM domain is found either by itself or in combination with TLDC motifs found in putative membrane-bound proteins (Zhang *et al.* 2009). The RED protein has a single LysM domain near the N terminal end of the protein. It lacks the transmembrane region found in most LysM proteins of bacteria, fungi, and plants, and, presumably, remains inside cells *vs.* on their surfaces.

In plants, some LysM proteins function in immune responses. Evidence in animals is mixed. Shi *et al.* (2013) reported that a red swamp crayfish gene carrying the LysM domain, PcLysM, shows increases in its mRNA accumulation when the crayfish are challenged with bacteria. Knockdown of PcLysM mRNA in the animals is accompanied by a decrease in the antimicrobial response. Laroche *et al.* (2013) reviewed two microarray studies that measured Zebrafish mRNA levels in response to challenges with bacteria. The studies failed to detect a change in the quantities of LysM domain containing mRNAs (Laroche *et al.* 2013). Four *D. melanogaster* genes contain a LysM domain, *CG15471*, *CG17985*, and *mustard* (*mtd*). Only *mtd* also contains a TLDC domain. It is the only *Drosophila* LysM-containing gene that has been investigated with respect to innate immunity. *mtd* has a mutant allele that increases fly tolerance to *Vibrio* infection and decreases the transcription of at least one antimicrobial peptide involved in innate immunity. However, the *mtd* transcript that is most influential in changing sensitivity lacks the LysM domain and carries a TLDC domain (Wang *et al.* 2012b).

The *CG12207* protein has two different N terminal sequences. Neither of these appears to be a signal sequence (Petersen *et al.* 2011). Like the *cho* and *ma* genes, *CG12207* is maternally deposited and is ubiquitously expressed. Its highest expression is observed in Malpighian tubules (Chintapalli *et al.* 2007; Hammonds *et al.* 2013; Tomancak *et al.* 2002, 2007). Two of the five gene interactions listed in the Flybase Interactions Browser for *CG12207* are with proteins related to trafficking: CG16817 influences Golgi organization and ZnT63C transports Zn (Guruharsha *et al.* 2011). The Golgi apparatus contributes cargo to the endosomes and lysosomes that may be involved in vesicular trafficking. *ZnT63C* moves Zn out of the cytoplasm either into intracellular compartments or outside the cell membrane (Kondylis *et al.* 2011; Wang *et al.* 2009). Another Zn transporter, *Catsup*, has been shown to disrupt trafficking of NOTCH, Epidermal Growth Factor Receptor, and *Drosophila* Amyloid Precursor-Like proteins (Groth *et al.* 2013).

### The MAH protein is predicted to be an amino acid transporter that is not essential

InterPro analysis of the MAH predicted protein identified 11 transmembrane helices and a conserved domain found in amino acid transporters (Mitchell *et al.* 2015). The NCBI Conserved Domain Database identified the domain in MAH as the Solute Carrier (SLC) families 5 and 6-like; solute binding domain. Two studies (Romero-Calderon *et al.* 2007; Thimgan *et al.* 2006) fail to place *CG13646* into the *D. melanogaster* SLC6 protein group and Romero-Calderon *et al.* (2007) suggest that the protein may be an amino acid permease.

Unlike *cho* and *ma*, *mah* is not essential because the predicted protein in the *mah/mah* fly is a truncated, and probably inactive enzyme, yet these flies are viable. The expression of *mah* is limited, being ranked as present in the larval central nervous system, adult eye, Malpighian tubule, and testes (Chintapalli *et al.* 2007). Its time of highest expression is the white prepupal stage when eye pigmentation begins (Graveley *et al.* 2011).

### Disruption of both types of pigments is a good criterion to identify genes involved in Drosophila vesicular trafficking

Four candidate genes were identified as possible granule group genes based on one characteristic, decreases in the amounts of both ommochromes and pteridines found in fly eyes. Of these, *ma* is the best example of such a gene since it is a member of the HOPS complex, like some previously identified granule group genes. The vesicular ATPase, *cho*, is also very important in vesicle maturation and function. The *mah* gene may well be important in transport to pigment granules given that it appears to be a membrane protein similar to other amino acid carriers. The *red* gene’s function and significance in transport are unknown.

*cho* and *ma* can be used to study the involvement of their products in a variety of processes when their proteins are expressed *in vivo*. These two genes are essential, but the mutants are visible and viable without partial RNAi knockdowns. Further, since the genes are ubiquitously expressed they could be tested in screens detecting changes in tissues and organs other than the eye. They can also be used in screens in which the tested genes are knocked down by RNAi. The use of mutants in whole organisms to understand the effects of genes and genetic interactions is very powerful.

Trafficking is a complex phenomenon in which many genes participate. A simple criterion allows the discovery of more *Drosophila* trafficking genes and more alleles of identified genes. There is a set of unmapped, eye color mutants that have had their relative pteridine and ommochrome levels tested that could be identified using the techniques of deletion mapping and sequencing. More genes/alleles could be discovered whose use would contribute to understanding vesicular trafficking.

## 

## Supplementary Material

Supplemental Material
