# Supplementary material for: An Eye on Trafficking Genes: Identification of Four Eye Color Mutations in Drosophila
Source: G3 (Bethesda). 2016 Aug 23;6(10):3185–96. doi: 10.1534/g3.116.032508 (PMC5068940; doi:10.1534/g3.116.032508)
Supplement: Supplemental Material [file supp_6_10_3185__index.html]

An Eye on Trafficking Genes: Identification of Four Eye Color Mutations in Drosophila — Supplemental Material 

# An Eye on Trafficking Genes: Identification of Four Eye Color Mutations in *Drosophila*

## Supplemental Material for Grant *et al.*, 2016

**Files in this Data Supplement:**

- Figure S1 - Alignments of the predicted CG13646 protein with proteins from insects and mammals. (.pdf, 279 KB)
- Figure S2 - Alignment of CG12207 sequences from Genbank, OreR (progenitor of *redK1*), *redK1* and *red1*. (.pdf, 417 KB)
- Table S1 - Deletion coordinates arranged by starting position. (.pdf, 350 KB)
- Table S2 - Primers used for sequencing and cloning *cho, ma, mah and red*. (.pdf, 288 KB)
- Table S3 - Sequence differences between the *Drosophila melanogaster* genome sequence and mutant alleles of *maroon, chocolate, and mahogany*. (.pdf, 380 KB)
- Table S4 - Identifiers for proteins used in alignments. (.pdf, 257 KB)
- Table S5 - Distribution of CG122207 and CG3259 substitutions in *red1*, *redK1* and OreR stocks. (.pdf, 347 KB)
