# Supplementary material for: An Eye on Trafficking Genes: Identification of Four Eye Color Mutations in Drosophila
Source: G3 (Bethesda). 2016 Aug 23;6(10):3185–96. doi: 10.1534/g3.116.032508 (PMC5068940; doi:10.1534/g3.116.032508)
Supplement: Supplemental Material [file supp_g3.116.032508_FigureS1.pdf]

A.

```
Dmel_gi|24649865|ref|NP_651316.1|G13646       1 MFFFRRQNAKPAPRPSRAPSFALPKRRPPVQLTPSHQHDQLPFLRRSRFSALPHFRAYED
Dsim_gi|195573673|ref|XP_002104816.1|         1 MFFFRRQNAKPAPRPSRAPSFALPKRRPPVQLTPSHQHDQLPFLRRSRFSALPHFRAYED
Dpseu_gi|198451685|ref|XP_001358480.2|        1 MFFFRRKNVKPAPRPSRAPSFALPKRRASVQLMPSHQHDQLPFLSRSRFSALPHFRAYED
Dvir_gi|194152331|gb|EDW67765.1|              1 MFFFRRSNVKPPPRPSRAPSFALPKRHSPVLLKPSHQHDQLPFLRRSRFSALPHFRAYED
Aedes_gi|157124582|ref|XP_001654116.1|        1 ------------------------------------------------------------
Flour_beetle_gi|642939900|ref|XP_0082002      1 MELS----------------------------------ERTLLL--------DHTASTKN
Silkworm_gi|512910840|ref|XP_004927269.1      1 MNGNCN----PE--------TPLE------------ESQTKPLVVSS-----HDRYTVGE
Waspgi|156549334|ref|XP_001601210.1|_1        1 MRED------------------------------------IPL-------------LLSR
Aphid_gi|641671950|ref|XP_008185768.1|        1 MI---------------------------------------------------------A
Louse_gi|212505775|gb|EEB10155.1|             1 MKDENYSGGSRRSRNPRDPIWTCVAGDPRIT---QQREYVVPSTSDS-----ESAPLASN
Louse_gi|242003872|ref|XP_002422893.1|        1 ------------------------------------------------------------
consensus                                     1 ...... . .. ...............  . . ........... ...............

Dmel_gi|24649865|ref|NP_651316.1|G13646      61 EPENLSLFIAILYVVDLFGIFPFVTLPALLVKLGYFGILLVLSIIILQIYTSFLLSQCWT
Dsim_gi|195573673|ref|XP_002104816.1|        61 EPENLSLFIAILYVVDLFGIFPFVTLPALLVKLGYFGVLLVLSIIILQIYTSFLLSQCWT
Dpseu_gi|198451685|ref|XP_001358480.2|       61 EPENLSLFIAILYVVDLFGIFPFVTLPAMLVELGYFGILLVLSIIFLQIYTSFLLSQCWT
Dvir_gi|194152331|gb|EDW67765.1|             61 EPESLSLFIAILYVVDLFGIFPFLTLPALLVRLGYFGLLLVLSIIFLQMYTSFLLSQCWT
Aedes_gi|157124582|ref|XP_001654116.1|        1 SSPNLSLFFASLCVIDLFGVFPIVALPKSIISCGLYGIPLVLLVITLQIYTATVLGRCWT
Flour_beetle_gi|642939900|ref|XP_0082002     19 CHNKLSVIFAIVCIVDVFGVFPVTTLPKAIIDCGIYGGIILCIVCSIQIYTAILLGKSWI
Silkworm_gi|512910840|ref|XP_004927269.1     32 TTGGLSVLFTILCIVDLFGVFPVIALPKSVISCGIYGIPLVLSVVGLQLYTAALLGRCWL
Wasp gi|156549334|ref|XP_001601210.1|_1      12 KDSGLSLLFATLCVVDIFGVFPIIALPRAIVQCGWLGIPLVFVVFGFQIYTASLLGKSWN
Aphid_gi|641671950|ref|XP_008185768.1|        4 DSSGLTVPFAILCIVDLFGIFPIVVLPGPIIKCG---IPLAIGVFAIQVYTAILLGKCWI
Louse_gi|212505775|gb|EEB10155.1|            53 HVKGLSVTSAAVFIAGEMAGSGVLALPRAVVD----------------------AGACWE
Louse_gi|242003872|ref|XP_002422893.1|        1 ----------------------------AVVD----------------------AGACWE
consensus                                    61 ... ........................ ............ ...........*.

Dmel_gi|24649865|ref|NP_651316.1|G13646     121 MAELLDPSIQQKRNYPYAALAELAYGPYMSLLVSVLLDLSIFAMAVPSVVMAAQNLEGVV
Dsim_gi|195573673|ref|XP_002104816.1|       121 MAELLDPSIQQKRNYPYAALAELAYGPYMSLLVSVLLDLSIFAMAVPSVVMAAQNLEGVV
Dpseu_gi|198451685|ref|XP_001358480.2|      121 MAEHLDPSILQKRNYPYAALAELAYGPYVSLLVSVLLDLSIFAMAVPSVVMAAENLEAVV
Dvir_gi|194152331|gb|EDW67765.1|            121 MAEYLDPSILQKRNYPYAALAELAYGPYMSLLVSVLLDLSIFAMAVPSIVMAAENLEAVV
Aedes_gi|157124582|ref|XP_001654116.1|       61 IAEKLDPSIVRKNRYPYAAIAEFTYGKRMSVFVTVLLDMTVFGGGIPNLLVASQNLQLLG
Flour_beetle_gi|642939900|ref|XP_0082002     79 IAEKIDVSVETKNRYPYSALAEITYGTTFSNFVSFLVNVTIFGGGIPNLIVASQNIELLG
Silkworm_gi|512910840|ref|XP_004927269.1     92 LAKEITPIISEKNRSPYAAVAQLAFGDPARRLVIFLIDATVFGSGVPNFILAAQSLQIFW
Wasp gi|156549334|ref|XP_001601210.1|_1      72 IANVVDPSISRKNRYPLAAVTELTLGRRASKWVAILLDLTVFAGGVPNLLVASYNLQLFG
Aphid_gi|641671950|ref|XP_008185768.1|       61 IAEEIEPNIVKKNRYPYAALAELIFGNKVKRIVTVMLDVAVFGACIPNLLIASYNLHILG
Louse_gi|212505775|gb|EEB10155.1|            91 ILEERYLDYRQPVRNPYATIAFRAVGPWARKLVSFCIQFTLFGAGTVYLLLAAQIVKDLL
Louse_gi|242003872|ref|XP_002422893.1|       11 ILEERYLDYRQPVRNPYATIAFRAVGPWARKLVSFCIQFTLFGAGTVYLLLAAQIVKDLL
consensus                                   121 ..............*.........*......*........*.........*.......

Dmel_gi|24649865|ref|NP_651316.1|G13646     181 LRMSAGQYNFSYCYWAIIVGLVICPLMWLGSPKHMRGLAIIAVCVMIVIVALLWFCLFAA
Dsim_gi|195573673|ref|XP_002104816.1|       181 LRMSAGQYNFSYCYWAIIVGLVICPLMWLGSPKHMRGLAIIAVCVMIVIVALLWFCLFAA
Dpseu_gi|198451685|ref|XP_001358480.2|      181 LRMSAGHYNFSYCYWAIIVGLVICPLMWLGSPKHMRGLAIIAVCVMILIVALLWFCLFAA
Dvir_gi|194152331|gb|EDW67765.1|            181 LRMSAGQYSFSYCYWAIVVGLVICPLMWLGSPKHMRGLAIMAACVLIIIVGLLWYCLLDA
Aedes_gi|157124582|ref|XP_001654116.1|      121 SKLTGGEFEFSFCYWLMLIGMFLCPIMWLGSPKNMRPLASISVIICSSVAFLTWLSIGED
Flour_beetle_gi|642939900|ref|XP_0082002    139 LRLSSDTFEVSFCYWIIIIGMILCPVLWLGSPKDMKLLCTISVANVVIVFLLVTECLIFS
Silkworm_gi|512910840|ref|XP_004927269.1    152 WKISGGNVGVTYCIWMLVLALLLCPIMWLGSPKDMKPLALTSVFIVTTVAVSTWTCIIQD
Wasp gi|156549334|ref|XP_001601210.1|_1     132 LKVSQMQFNLSFCYWLLVVGVLLCPVMWLGSPRDMKWVVTISVCAVSATAVLIWWSIVHD
Aphid_gi|641671950|ref|XP_008185768.1|      121 IKLSSERFDVSPCIWLIVIGIILCPPLWLGSPKDMKWIVSSSVFFVGSVSVLTWIAMYDT
Louse_gi|212505775|gb|EEB10155.1|           151 DDY---FPNFGLCIWFLIISIILMPAMWFGSPKDFRVVGIGALLTTAIACVLIFTQIVLD
Louse_gi|242003872|ref|XP_002422893.1|       71 DDY---FPNFGLCIWFLIISIILMPAMWFGSPKDFRVVGIGALLTTAIACVLIFTQIVLD
consensus                                   181 ...........*.*.........*..*.***......................... .

Dmel_gi|24649865|ref|NP_651316.1|G13646     241 PAIGTP-FEGISLELPGFLTVLNSYSILAFQFDIHPVLLTLQIDMKQKSQVSWAALIGIA
Dsim_gi|195573673|ref|XP_002104816.1|       241 PAIGTP-FEGISLELPGFLTVLNSYSILAFQFDIHPVLLTLQIDMKHKSQVSWAALIGIA
Dpseu_gi|198451685|ref|XP_001358480.2|      241 PAIGTP-FEGISMELPGFLTVLSSYSILAFQFDIHPVLLTLQIDMKRKSQVSWAATIGIA
Dvir_gi|194152331|gb|EDW67765.1|            241 AAIGAP-FQGISMELPGFLTILSSYSILAFQFDIHPMLLTLQIDMKRKSQVSWAALVGIA
Aedes_gi|157124582|ref|XP_001654116.1|      181 TFVTNP-FKGIELGTPSWIRLLKAYGIIAFQFDIHPMLLTIQVDMEKKRKIGKAVFLGLM
Flour_beetle_gi|642939900|ref|XP_0082002    199 SRTPTTTDDSFQTEQSFWELMFICYGIISFQFDIHPSILTIQVDMREKSKLNSAVLGGFA
Silkworm_gi|512910840|ref|XP_004927269.1    212 DVSPVS-TGTILEYQPHAPDFLIAYGILAFQFDIHPMLLTIQVDMRDSKKVNKAVLGGFA
Wasp gi|156549334|ref|XP_001601210.1|_1     192 QEPMNY---SPVPTSPSWDTFISGYGMLAFQFDVHPTIMTVQVDMKKPKDINKAVIFSFM
Aphid_gi|641671950|ref|XP_008185768.1|      181 QREIY-----APIPEPSWNSVALAYGLLAFQFDVHPLVLTVQMDMVDKRKLPVAIICAFL
Louse_gi|212505775|gb|EEB10155.1|           208 GLHNM---KPVKRKVHGFYDFFVSFGTILFAFGGASTFPTIQNDMINKEKFSKSVFIAFS
Louse_gi|242003872|ref|XP_002422893.1|      128 GLHNM---KPVKRKVHGFYDFFVSFGTILFAFGGASTFPTIQNDMINKEKFSKSVFIAFS
consensus                                   241  ..... ......................*.*.......*.*.**..............
```

```
Dmel_gi|24649865|ref|NP_651316.1|G13646      300 ITCSVAIFGSIIAAYKFGSMIADNLLQSLPTSVPFYVMLILMALQLCFSVTVASSAMFMQ
Dsim_gi|195573673|ref|XP_002104816.1|        300 ITCSVAIFGSIIAAYKFGSMIADNLLQSLPTSVPFYVMLILMALQLCFSVTVASSAMFMQ
Dpseu_gi|198451685|ref|XP_001358480.2|       300 ITCSVAIFGSIIAAYKFGSMIASNLLQSLPTSVPFYVMLILMSLQLCFSVTVASSAMFLQ
Dvir_gi|194152331|gb|EDW67765.1|             300 ITCSVAIVGAIIAAYKFGALIASNLLQSLPTSVPFYVMLILMSLQLCFSVTVAGSAMFLQ
Aedes_gi|157124582|ref|XP_001654116.1|       240 ATCTLSTVTTVFAAYRYGMDTTNNVLQILPKSWPLYITILLVTLQLCLSSAVGNSALFQH
Flour_beetle_gi|642939900|ref|XP_0082002     259 ISLGMFSIVTALAASKYGLSVKPSLLETLPTTIPLHFAALFVALQLCLSSAVSNSALYQY
Silkworm_gi|512910840|ref|XP_004927269.1     271 ITGFMFTTTAFLVATRYGQDVTNNILQTIPPSIPLYLVALLVTLQLCLSSAVSNSALFQH
Wasp gi|156549334|ref|XP_001601210.1|_1      249 ISGTLFAVTAGLAVWRYGGNTSTNILQVMPPGIMVQTAILISAVQLCLSSAIGHSALFQH
Aphid_gi|641671950|ref|XP_008185768.1|       236 ITCSLFLITTVIGYVRFGSLLSSNLLDQLSNSYILDVNITLVTIQICLSTAVSTTALFQH
Louse_gi|212505775|gb|EEB10155.1|            265 VILGLYVPVTFGGYIVYGEMVTPNIILSLGHTSLVKMANILMAIHLVLAFLIVINPVCQE
Louse_gi|242003872|ref|XP_002422893.1|       185 VILGLYVPVTFGGYIVYGEMVTPNIILSLGHTSLVKMANILMAIHLVLAFLIVINPVCQE
consensus                                    301 ........ ........*.........................................

Dmel_gi|24649865|ref|NP_651316.1|G13646      360 IENYFKLPESLSFKRMLIRSSVLALEVLVAEFVPSFDALMDVVGGTITGPLVFILPPLLY
Dsim_gi|195573673|ref|XP_002104816.1|        360 IENYFKLPESLSFKRMLIRSSVLALEVLVAEFVPSFDALMDVVGGTITGPLVFILPPLLY
Dpseu_gi|198451685|ref|XP_001358480.2|       360 IENYFKLPESLSLKRMAIRSTVLALEVLVAEFVPSFDALMDVVGGTITGPLVFILPPLLY
Dvir_gi|194152331|gb|EDW67765.1|             360 IENFFKLPESLSCKRILIRSGVLALEVLVAEFVPSFDTLMDVVGGTITGPLVFILPPLLY
Aedes_gi|157124582|ref|XP_001654116.1|       300 VEDVLGASRDFTIKRCVIRSSLVWLAVLIAELLPRFDVVMGIIGGTLTGPLIFILPPLFY
Flour_beetle_gi|642939900|ref|XP_0082002     319 MEDCMSISPAFNHRRCVLRTTLTFLAVLIAESVPRFDLVMSLIGGTLTGPLIFIFPPLFY
Silkworm_gi|512910840|ref|XP_004927269.1     331 IEDLLQIPRNFCIQRCLIRSSVVAVAVFLAETVPRFDLVMGLVGSTLTGPLMFIFPPLFF
Wasp gi|156549334|ref|XP_001601210.1|_1      309 LEDQLRVDSSFSWKRCATRSAIVFLGVALGESVPRFDIVMSLIGGTLVGPLVFVLPPLMY
Aphid_gi|641671950|ref|XP_008185768.1|       296 IEHFLKIPKEFNRRRCVLRSCIVMLAVTIGEAVPRFDLLMGLVGALLTGPLMFLLPPLFY
Louse_gi|212505775|gb|EEB10155.1|            325 LEEHFKIPMDFGIKRCLIRSGIMLTMVFVGETIPRFRKILALVGGSTITLLTFVFPALFY
Louse_gi|242003872|ref|XP_002422893.1|       245 LEEHFKIPMDFGIKRCLIRSGIMLTMVFVGETIPRFRKILALVGGSTITLLTFVFPALFY
consensus                                    361 .*............*...*.......*...*..*.*.......*......*.*..*.*..

Dmel_gi|24649865|ref|NP_651316.1|G13646      420 RRIRRMERVHQRIAA-EASYGSL-------------------------------------
Dsim_gi|195573673|ref|XP_002104816.1|        420 RRIRRMERVHQRIAA-EASYGSL-------------------------------------
Dpseu_gi|198451685|ref|XP_001358480.2|       420 RRIRRMERVHQRIAA-EASYGSL-------------------------------------
Dvir_gi|194152331|gb|EDW67765.1|             420 RRIRRMERVHQRIAA-EASYGSL-------------------------------------
Aedes_gi|157124582|ref|XP_001654116.1|       360 QKMISLEAIYYQEMERIQSRDT--------------------------------------
Flour_beetle_gi|642939900|ref|XP_0082002     379 LKMLSMENKQMKQMA-METFTS--------------------------------------
Silkworm_gi|512910840|ref|XP_004927269.1     391 LKLCYLKSKKDITES-QNAYSN--------------------------------------
Wasp gi|156549334|ref|XP_001601210.1|_1      369 SKARALCSASLRRTS-APEYLCSGPERRAGLAARELFADPRVHSRSTHFGFRYGDEDKND
Aphid_gi|641671950|ref|XP_008185768.1|       356 IKIRSLRRLKIKKSE-GVCYRTF-------------------------------------
Louse_gi|212505775|gb|EEB10155.1|            385 MLLCRQHKLEWPERS-IPL-----------------------------------------
Louse_gi|242003872|ref|XP_002422893.1|       305 MLLCRQHKLEWPERS-IPL-----------------------------------------
consensus                                    421 ............... .......

Dmel_gi|24649865|ref|NP_651316.1|G13646      442 ----------------------------------PLD-LNYDPVDLEMEPLLVISP----
Dsim_gi|195573673|ref|XP_002104816.1|        442 ----------------------------------PLD-LNYDPVELEMEPLLVTSP----
Dpseu_gi|198451685|ref|XP_001358480.2|       442 ----------------------------------PLD-LNYDPVELEMEPLLVAAK----
Dvir_gi|194152331|gb|EDW67765.1|             442 ----------------------------------PLD-LNYDPIELEMEPLLVVAK----
Aedes_gi|157124582|ref|XP_001654116.1|       382 ---------------------------------LPLFPSDYGSIGPQGTDTRRPPP----
Flour_beetle_gi|642939900|ref|XP_0082002     400 ---------------------------------VVYS-RNEEEKILT-------------
Silkworm_gi|512910840|ref|XP_004927269.1     412 ---------------------------------NATN-GSLDSQKSKLNKIETSGSSQQN
Wasp gi|156549334|ref|XP_001601210.1|_1      428 GYYFTYYEDEDQVEVNPYTESVECVFNDDSGARKKQS-DDLELSRSGSLPVMAD------
Aphid_gi|641671950|ref|XP_008185768.1|       378 ----------------------------------PNA-KLT-------------------
Louse_gi|212505775|gb|EEB10155.1|            403 ------------------------------------------------------------
Louse_gi|242003872|ref|XP_002422893.1|       323 ------------------------------------------------------------
consensus                                    481                                   ... ...............

Dmel_gi|24649865|ref|NP_651316.1|G13646      463 -------------------------------PTTPRGCWLRFVRLLHRLECDVSCTMAVL
Dsim_gi|195573673|ref|XP_002104816.1|        463 -------------------------------PTTPRGCWLRLLRFLHRLECDVSCTMAVL
Dpseu_gi|198451685|ref|XP_001358480.2|       463 -------------------------------PNSPRGCWLRLVRLLHRLDCDVSCTMGVL
Dvir_gi|194152331|gb|EDW67765.1|             463 -------------------------------PHTTQGCWLRLVRLLHRLECDISCTMGVL
Aedes_gi|157124582|ref|XP_001654116.1|       405 -------------PTL--HW----------MGDCFSICHDRFQQFCRFLYSDCILSGAVI
Flour_beetle_gi|642939900|ref|XP_0082002     413 ---------------------------------NTDECQFEITKSILGAKLDIVCCVTII
Silkworm_gi|512910840|ref|XP_004927269.1     438 GVVTVIQNGENVNPSLYTKY----------KTFSRDYAEMGKEDEYTIKWYDIVLALVVM
Wasp gi|156549334|ref|XP_001601210.1|_1      481 -------------------ASRPSVRPPRRQEALQNPNANRPRNFCTKQRIIDWFGYCVA
Aphid_gi|641671950|ref|XP_008185768.1|       384 ---------------------------------------------PIVGFKRLALLIFII
Louse_gi|212505775|gb|EEB10155.1|            403 --------------------------------------------------HIRLYLWELI
Louse_gi|242003872|ref|XP_002422893.1|       323 --------------------------------------------------HIRLYLWELI
consensus                                    541                                . .  ........ ...............
```

```
Dmel_gi|24649865|ref|NP_651316.1|G13646      492 IFGLLATFLSTYLNIFSLASLF-TNNSPCLSNLTKHF-----------------------
Dsim_gi|195573673|ref|XP_002104816.1|        492 IFGLLATFLSTYLNIFSLSSLF-TNNSPCLSNLTKHF-----------------------
Dpseu_gi|198451685|ref|XP_001358480.2|       492 LFGILATFLSTYLNLFSLPSLF-TNNSPCLSNLTR-P-----------------------
Dvir_gi|194152331|gb|EDW67765.1|             492 IFGLLATFLSTYLNLFTLADLF-KNNSPCLSNLTA-H-----------------------
Aedes_gi|157124582|ref|XP_001654116.1|       440 LFGIGATLISTYYNIFDVKDIGTQFWGSCAANITLVS-------D---------------
Flour_beetle_gi|642939900|ref|XP_0082002     440 TLSAVCTVITTYVNIRNAIAFV-NFTQPCIFNVSSSL------IS---------------
Silkworm_gi|512910840|ref|XP_004927269.1     488 TLGIAATVIATYCSWAHSIEYA-EFSPPCLLNATIAA------RS---------------
Wasp gi|156549334|ref|XP_001601210.1|_1      522 FTGILITVSSTYINIRNTIRYV-QFIPPCIVNATANI------PE---------------
Aphid_gi|641671950|ref|XP_008185768.1|       399 LAGTLATVLSTVSGIRDTIIYA-KFTPSCIMKLFECYWLIILGVALCPLTWLGSPKDTKW
Louse_gi|212505775|gb|EEB10155.1|            413 IIGVIGGTASSYSAILSIFSPD-AFSLPCYWIFS--------------------------
Louse_gi|242003872|ref|XP_002422893.1|       333 IIGVIGGTASSYSAILSIFSPD-AFSLPCYWIFS--------------------------
consensus                                    601 ......................  ....*.....

Dmel_gi|24649865|ref|NP_651316.1|G13646          ------------------------------------------------
Dsim_gi|195573673|ref|XP_002104816.1|            ------------------------------------------------
Dpseu_gi|198451685|ref|XP_001358480.2|           ------------------------------------------------
Dvir_gi|194152331|gb|EDW67765.1|                 ------------------------------------------------
Aedes_gi|157124582|ref|XP_001654116.1|       478 ---------------------------------------L--------
Flour_beetle_gi|642939900|ref|XP_0082002     478 ---------------------------------------L--------
Silkworm_gi|512910840|ref|XP_004927269.1     526 -------------FLES-------------------QMAL--------
Wasp gi|156549334|ref|XP_001601210.1|_1      560 -------------LG---------------------------------
Aphid_gi|641671950|ref|XP_008185768.1|       458 ISVFSSFFVIVCAFLTWFCMLSDDDTKITPHDLFHFHLGTEYPLLLVY
Louse_gi|212505775|gb|EEB10155.1|                ------------------------------------------------
Louse_gi|242003872|ref|XP_002422893.1|           ------------------------------------------------
consensus                                    661
```

B.

```
vesicu_GABA_mel       1 MSFIAKLKATPLPPLRNILNVAVQTARQQIPERKDYEQPPGSTAQQHHHSQQAQHKAMEA
vesic_GABA_Mus        1 MATLLRS-------------------------------KLTNVATSVSNKSQAKVSGMFA
SLC32A1_Homo_sapien   1 MATLLRS-------------------------------KLSNVATSVSNKSQAKMSGMFA
CG13646=Dmel          1 MFFFRRQ-------------------------------NAKPAPRPSRAPSFA-LPKRRP
consensus             1 *. ....                               ..............*.......

vesicu_GABA_mel      61 GMDGGDTTEMSSNPFRNAGSWTNDGEGGGDGDGE-----YRNEYQSTSFNEYDGRYQQTD
vesic_GABA_Mus       30 RMGFQAATDEEAVGFAHCDDLDFEHRQGLQMDILKSEGEPCGDEGAEAPVEGDIHYQRGG
SLC32A1_Homo_sapien  30 RMGFQAATDEEAVGFAHCDDLDFEHRQGLQMDILKAEGEPCGDEGAEAPVEGDIHYQRGS
CG13646=Dmel         29 PVQLTPSHQHDQLPFLRRSRFSAL------------------------------------
consensus            61 ............. *.................... .......................

vesicu_GABA_mel     116 GFRQGSIASEGS-SFVCEGEGGGGCKIDEFQAAWNVTNAIQGMFIVSLPFAVLHGGYWAI
vesic_GABA_Mus       90 APLPPSGSKDQAVGAGGEFGGHDKPKITAWEAGWNVTNAIQGMFVLGLPYAILHGGYLGL
SLC32A1_Homo_sapien  90 GAPLPPSGSKDQVGGGGEFGGHDKPKITAWEAGWNVTNAIQGMFVLGLPYAILHGGYLGL
CG13646=Dmel         53 --------------PHFRAYEDEPENLSLFIAILYVVDLFGIFPFVTLPALLVKLGYFGI
consensus           121 .   .. ...  ...................*...*...........**......**...

vesicu_GABA_mel     175 VAMVGIAHICCYTGKVLVQCLYEPDPATGQ-MVRVRDSYVAIAKVCFGP---KLGARAVS
vesic_GABA_Mus      150 FLIIFAAVVCCYTGKILIACLYEEN-EDGE-VVRVRDSYVAIANACCAPRFPTLGGRVVN
SLC32A1_Homo_sapien 150 FLIIFAAVVCCYTGKILIACLYEEN-EDGE-VVRVRDSYVAIANACCAPRFPTLGGRVVN
CG13646=Dmel         99 LLVLSIIILQIYTSFLLSQCWTMAELLDPSIQQKRNYPYAALAELAYGP---YMS-LLVS
consensus           181 ..... .....**...*. *..... .... .......*.*.*.....*.........*

vesicu_GABA_mel     231 IAQLIELLMTCILYVVVCGDLLA-----GTYPQGSFDSRSWMLFVGIFLLPMGFLKSLKM
vesic_GABA_Mus      208 VAQIIELVMTCILYVVVSGNLMY-----NSFPGLPVSQKSWSIIATAVLLPCAFLKNLKA
SLC32A1_Homo_sapien 208 VAQIIELVMTCILYVVVSGNLMY-----NSFPGLPVSQKSWSIIATAVLLPCAFLKNLKA
CG13646=Dmel        155 VLLDLSIFAMAVPSVVMAAQNLEGVVLRMSAGQYNFSYCYWAIIVGLVICPLMWLGSPKH
consensus           241 ..............**.......     .... .. ....*...  ....*...*. .*.

vesicu_GABA_mel     286 VSTLSFWCTMSHIVINAVILGYCLLQIGDWGWSKVRFSIDMENFP---ISLGVIVFSYTS
vesic_GABA_Mus      263 VSKFSLLCTLAHFVINILVIAYCLSRARDWAWEKVKFYIDVKKFP---ISIGIIVFSYTS
SLC32A1_Homo_sapien 263 VSKFSLLCTLAHFVINILVIAYCLSRARDWAWEKVKFYIDVKKFP---ISIGIIVFSYTS
CG13646=Dmel        215 MRGLAIIAVCVM-IVIVALLWFCLFAAPAIGTPFEGISLELPGFLTVLNSYSILAFQFDI
consensus           301 ... ..................**............. .....*.   .*.....*....
```

```
vesicu_GABA_mel       343 QIFLPTLEGNMIDRSKFNWMLDWSHIAAAVFKAGFGYICFLTFQNDTQQVITNNLHSQGF
vesic_GABA_Mus        320 QIFLPSLEGNMQQPSEFHCMMNWTHIAACVLKGLFALVAYLTWADETKEVITDNLPGS-I
SLC32A1_Homo_sapien   320 QIFLPSLEGNMQQPSEFHCMMNWTHIAACVLKGLFALVAYLTWADETKEVITDNLPGS-I
CG13646=Dmel          274 HPVLLTLQIDMKQKSQVSWAALIGIAITCSVAIFGSIIAAYKFGSMIADNLLQSLPTS-V
consensus             361 ...*..*...*...*... ...................................*... .

vesicu_GABA_mel       403 KGMVNFFLVIKAL-LSYPLPYYAACELLERNFFRGPPKTKFPTIWNLDGELKVWGLGFRV
vesic_GABA_Mus        379 RAVVNLFLVAKAL-LSYPLPFFAAVEVLEKSLFQEGSRAFFPACYGGDGRLKSWGLTLRC
SLC32A1_Homo_sapien   379 RAVVNIFLVAKAL-LSYPLPFFAAVEVLEKSLFQEGSRAFFPACYSGDGRLKSWGLTLRC
CG13646=Dmel          333 PFYVMLILMALQLCFSVTVASSAMFM-------------QIENYFKLPESLSFKRMLIRS
consensus             421 ...*...*....* .*......*......................  ...*.......*.

vesicu_GABA_mel       462 GVIVSTILMAIFIPHFSILMGFIGSFTGTMLSFIWPCYFHIKIKGHLLDQKEIA------
vesic_GABA_Mus        438 ALVVFTLLMAIYVPHFALLMGLTGSLTGAGLCFLLPSLFHLRLLWRKLLWHQVF------
SLC32A1_Homo_sapien   438 ALVVFTLLMAIYVPHFALLMGLTGSLTGAGLCFLLPSLFHLRLLWRKLLWHQVF------
CG13646=Dmel          380 SVLALEVLVAEFVPSFDALMDVVGGTITGPLVFILPPLLYRRIRRMERVHQRIAAEASYG
consensus             481 .......*.*...*.*..**...*......*.*..*.................

vesicu_GABA_mel       516 ----------------------------------------------KDYLIIGLGVLFGV
vesic_GABA_Mus        492 ----------------------------------------------FDVAIFVIGGICSV
SLC32A1_Homo_sapien   492 ----------------------------------------------FDVAIFVIGGICSV
CG13646=Dmel          440 SLPLDLNYDPVDLEMEPLLVISPPTTPRGCWLRFVRLLHRLECDVSCTMAVLIFGLLATF
consensus             541                                               ........*.....

vesicu_GABA_mel       530 IGIYDSGNALINAFEIGLP--------F
vesic_GABA_Mus        506 SGFVHSLEGKFAGLET------------
SLC32A1_Homo_sapien   506 SGFVHSLEGLIEAYRTNAE--------D
CG13646=Dmel          500 LSTYLNIFSLASLFTNNSPCLSNLTKHF
consensus             601 ... ....... ..... .        .
```

Figure S1 Alignments of the predicted CG13646 protein with proteins from insects and mammals. The consensus sequence is given in the bottom line for each amino acid group. If a position is conserved in all members an asterisk appears in the consensus. Residues identical to the consensus are shaded black. Residues similar to the consensus are labeled in gray. **A.** An alignment of predicted insect orthologs of the CG13646 protein. The first four sequences are from Drosophila species. As the shading indicates these show very high similarity to each other. While regions of complete consensus occur, the sequences from a non-Drosophila species show considerable deviation from the Drosophila group. The symbols represent *D. melanogaster* (CG13646=Dmel), *D. simulans* (Dsim), *D. pseudoobscura* (D.pseudoobs), *D. virilis* (Dvirilis), *Aedes aegypti*, Red flour beetle (*Tribolium casteneum*), Chinese Silkworm (*Bombyx_mori*), parasitic wasp (Nasonia_vitripennis), Pea aphid (*Acyrthosiphon pisum*), and two partial sequences from the human body louse (*Pediculus humanus corporis*). Gene identifiers are in Supplemental Table 4. **B.** Comparisons of the predicted CG13646 protein with the vesicular GABA transporters from *Drosophila* (vesicu_GABA_mel), *Mus musculus* (vesic_GABA_Mus) and *Homo sapien* (SLC32A1_Homo_sapien). Note that while PSI-Blast searches of mammals using CG13646 recover the GABA vesicular transporters from mouse and human, the *Drosophila*

ortholog of GABA vesicular transporter shows greater sequence similarity to the *Mus* and *Homo* proteins than CG13646 does.
