# Supplementary material for: An Eye on Trafficking Genes: Identification of Four Eye Color Mutations in Drosophila
Source: G3 (Bethesda). 2016 Aug 23;6(10):3185–96. doi: 10.1534/g3.116.032508 (PMC5068940; doi:10.1534/g3.116.032508)
Supplement: Supplemental Material [file supp_g3.116.032508_FigureS2.pdf]

```
Genbank    61[1]  ATGCCATTCTTCAATTCCAGCCGAAGAAACCGAAAGCCGAGAGCCCAGCTGAACGCCCAA  120
OreR       61     ....................................................G.......  120
redK1      61     ....................................................G.......  120
//
Genbank    241    CTGCTGCGCTCTTAGTTGTTTTGGCTAAGTTCACTTAGTTAGTGCCTTCGTTTAGCGGCG  300
OreR       241    ............................................................  300
redK1      241    ............................................................  300
red1       1      ..............................................C.............  60
//
Genbank    361    CGCTTTTGTATCTGTGTTTGTGAGTGCCgaaaagatgaatatatagaaatacacacaaat  420
OreR       361    ............................................................  420
redK1      361    ............................................................  420
red1       121    ..........C......................C..........................  180
//
Genbank    481    TTAAGTTAGAGAACGGTTACTGTCGTTGCCATGGGACAAACGTAAAGCCTCGATTGTGTG  540
OreR       481    .....................................G......................  540
redK1      481    .....................................G......................  540
red1       241    .....................................G......................  300
//
Genbank    1441   CACAACCTCGTTTGATGGCAGCCACCGaaaaaaaaaaCCAAGAAACATGTACCAAGAAAT  1500
OreR       841    ............................................................  900
redK1      1442   .......................M[2]....................................  1501
red1       1472                                  .............................  1500
//
Genbank    1501   CTCGACaaaaaaaaaaaTACCCACAGCGTTTAATTGTTTCATAATTGCGGAGTGAATTCA  1560
OreR       901    ......-.....................................................  959
redK1      1502   ......--....................................................  1559
red1       1501   ......--....................................................  1558

Genbank    1561   GCTGATAACGGGACAGACAAAAACGAAAATGAACCAAAGTTAATTGCTATCAGTGAGTGC  1620
OreR       960    ............................................................  1019
redK1      1560   ............................................................  1619
red1       1559   A...........................................................  1618

Genbank    1621   AGTGACCGTACTTAGTATATTTTATGTTCTTCGATGCAGGCGATCCAGTGTGAAAAGCAG  1680
OreR       1020   ...................................K........................  1079
redK1      1620   ............................................................  1679
red1       1619   ...................................T........................  1678
//
Genbank    1741   GCATTCGCAGGTGAGAAATCTAGTTAATTATACTATTAGTTAGAAAGGTAAGCACTCAAG  1800
OreR       1140   ........................................................Y...  1199
redK1      1740   ............................................................  1799
red1       1739   ........................................................T...  1798
//
Genbank    9181   CATTGCAGCACGTAGTAAACAGAAAATCACAATTGGATCGAAAAATCGCAAATTGCCATA  9240
OreR       1263   ..C..A......................................................  1322
redK1      9181   ..C..A......................................................  9240
red1       9179   ..C.........................................................  9238

Genbank    9241   AAACAGAGCACTCGCGTGTGCAAATATTAGCGCAATTCGTTGGCAGGCTGAGCAGTGGGC  9300
OreR       1323   ................................................C...........  1382
redK1      9241   ................................................C...........  9300
red1       9239   ................................................C...........  9298
```

---

[1] Genbank sequence position 61 corresponds to chr3R: 14,289,131.

[2] Additional nucleotide symbols include M=A or C, K=G pr T and Y=C or T.

```
Genbank     9301   GGTAGAGACGCCAACCCACCCGCTCCCACTCTCTCCGTCGCGCCGTTTCGCTCGCTCTGT  9360
OreR        1383   .............C..............................................  1442
red^K1      9301   .............C..............................................  9360
red^1       9299   ............................................................  9358

Genbank     9361   TTTGAAAGGCTGCCATGGGTGTGTGCGTGCGTGTGTGAGTTTGGGGCTCTGCGGCGGAG  9420
OreR        1443   ..............................T.............................  1502
red^K1      9361   ..............................T.............................  9420
red^1       9359   ..............................T.............................  9418
//
Genbank     9481   AGTGCGCCGCATAATTAATTATTGCGGAAATTAGTCGCTTTTCACTATAGTCGCCCCAAC  9540
OreR        1563   ..............................................T.............  1622
red^K1      9481   ..............................................T.............  9540
red^1       9479   ..............................................T.....T.......  9538

Genbank     9541   ACTGCGTTTAAAATCAGTTTTTGTGTAACAAAATTACAATTACATACATACATATATGTG  9600
OreR        1623   ....................C....................T............C.....  1682
red^K1      9541   ....................C....................T............C.....  9600
red^1       9539   .........................................T............C.....  9598

Genbank     9601   TTCGTATTGGGACTGTGCCTGTGTGCGTTGCGTGTTGGCCATGACATCACTACAATAAAT  9660
OreR        1683   .A.............A............................................  1742
red^K1      9601   .A.............A............................................  9660
red^1       9599   .A.............A....................................T.......  9658

Genbank     9661   ATAATACATTAATACAACAACCAAACAACTGAATCGAATCATTCTGAAATCCATTGCAGT  9720
OreR        1743   ............................................................  1802
red^K1      9661   ............................................................  9720
red^1       9659   .........................G..................................  9718
//
Genbank     9781   TCCGGGACTCCGGGCGCACCCTCAAGCGCTACGGGAGCACCTGCTGCAACAGCCTCCGGA  9840
OreR        1863   ............................................................  1922
red^K1      9781   .T..........................................................  9840
red^1       9779   .T..........................................................  9838

Genbank     9841   ACAATGAGACCCTCATCCGGCACATCGTCGAGAAGACGGACACGCTGCAGGGCATCGCCC  9900
OreR        1923   ............................................................  1982
red^K1      9841   ..................................................A.........  9900
red^1       9839   ............................................................  9898
//
Genbank    11281   AGCGCTTCAATTGCGCGTAATTGATGAGCTCCACCAGCTTATAAATATATCCCGTGCATT  11340
OreR        2066                          ......................................  2103
red^K1     11307                            ...............................T...  11341

Genbank    11341   GTATTTCAGACGGAGCAAATAAGACGGGCCAACCGCCTCTTCGCCTCGGACAGCCTGTTC  11400
OreR        2104    ............................................................  2163
red^K1     11342   ............................................................  11401
red^1      11340   ..............................C.............................  11399
//
Genbank    11461   GGCGATTCGATAGCCGCTTTCGACGCCGTGCTGGCCACGCCACCGGGCACTCCGGATGCC  11520
OreR        2224    .................Y..........................................  2283
red^K1     11462   ............................................................  11521
red^1      11460   .................C..........................................  11519
//
Genbank    11821   AGCAAGAGCCTGGACTCGGTGGCCGCAATGACGCCCGAGGAGGAGAACAGGAAATGCATG  11880
OreR        2584    .................K..........................................  2643
red^K1     11822   ............................................................  11881
red^1      11820   .................T..........................................  11879
```

Genbank	11881	AACGACTTCCTTAACAAGATAGACAACACCATTTCTGAGTCGCGCAAATATGTGGAGCGC	11940
OreR	2644	....R..................	2703
red^{K1}	11882	...........C..	11941
red^{1}	11880	....A..................	11939
Genbank	11941	TCTAAGGAGTAAGTATTTCCACCACTCAAAAGAGACCCAATCAATCAACATTTAAGATCA	12000
OreR	2704	....	2763
red^{K1}	11942	....	12001
red^{1}	11940	....G.................	11999
Genbank	12001	TTCAGCTGTCAATATTGGTATTTAAATCAATTAATCATAATTTGATAAGCTGACAGAACC	12060
OreR	2764	....	2823
red^{K1}	12002	....	12061
red^{1}	12000	....	12059
Genbank	12061	AATAGAAACTGGAAGTGATAGGTGATGAAGTGATGAACGAGTCGCTAGCTTGTAGAATTC	12120
OreR	2824	........T...	2883
red^{K1}	12062	........T...	12121
red^{1}	12060	....	12119
//			
Genbank	12421	CTGCGGGACGCAATCTAGCCTAATTACGCAGAGAGTTAATTTTACAATTCGTTTTTAATT	12480
OreR	3184	....	3243
red^{K1}	12420	....T..........	12479
//			
Genbank	12541	TAAATGTAATATAAATAATGATATCTATCGACAAATATGCGCGATACCTT	12590
OreR	3304	....	3353
red^{1}	12540	C...	12589

Figure S2 Alignment of CG12207 sequences from Genbank, OreR (progenitor of *red*^{K1}), *red*^{K1} and *red*^{1}. Nucleotides marked in blue are variants from one or more of the last three sequences that are found in the *Drosophila* Genome Reference Panel[3]. Nucleotides marked in green are variants that are absent from the Panel. Only sequence regions that contain changes in nucleotides for the *red* alleles are included in the figure. Breaks in sequence continuity are indicated by //.

[3] Mackay, T. F., S. Richards, E. A. Stone, A. Barbadilla, J. F. Ayroles *et al*, 2012 The *Drosophila melanogaster* genetic reference panel. Nature **482:** 173-178
