# Supplementary material for: An Eye on Trafficking Genes: Identification of Four Eye Color Mutations in Drosophila
Source: G3 (Bethesda). 2016 Aug 23;6(10):3185–96. doi: 10.1534/g3.116.032508 (PMC5068940; doi:10.1534/g3.116.032508)
Supplement: Supplemental Material [file supp_g3.116.032508_TableS1.pdf]

Table S1 Deletion coordinates arranged by starting position.

Gene/Deletion	DNA region removed
chocolate	
Df(1)BSC834	X:3,288,956..3,845,727
Df(1)ED6716	X:3,799,196..4,204,584
P{XP}^{d03180} and *PBac{RB}VhaAC39-1*^{e04316}	X:3,811,573-3,880,603
P{XP}ec^{d00965} and *PBac{WH}f06086*	X:3,836,070 -3,931,540
PBac{RB}VhaAC39-1^{e04316}and *P{XP}yin*^{d02176}	X:3,868,554-3,880,603
Df(1)BSC 877	X:3,928,381..4,324,781—4,430,748
Region containing *cho*	X:3,880,603-3,928,381
maroon	
Df(3R)ED5339	3R:9,227,076-3R:9,352,375
Df(3R)BSC507	3R:9,253R:9,246-3R:9,394,580
PBac{RB}Aats-trp^{e00999} and *P{XP}d00816*	3R:9,266,559-3R:9,394,620
Df(3R)Exel9036	3R:9,327,275- 3R:9,340,006
Region containing *ma*	3R: 9,226,559-9,327,275 or 3R:9,340,006-9,352,375
mahogany	
Df(3R)Exel6200	3R:24,769,342- 24,894,707
Df(3R)BSC318	3R:24,876,200- 3R:25,071,295
P{XP}CG31121^{d06890} and *PBac{WH}f01730*	3R:24,894,700- 24,945,164
Df(3R)BSC494	3R:25,003,409- 25,137,846
Region containing *mah*	3R:24,945,164-25,003,409
red Malpighian tubules	
Df(3R)Exel7321	3R:14,125,489..14,278,204
Df(3R)Exel6267	3R:14,277,936..14,481,841

Gene/Deletion	DNA region removed
red, cont.	
P{XP}trx^{d08983} and *PBac{RB}su(Hw)*^{e04061}	3R:14,283,990-3R:14,307,955
Region containing *red*	3R:14,283,990-14,307,955
