# Supplementary material for: An Eye on Trafficking Genes: Identification of Four Eye Color Mutations in Drosophila
Source: G3 (Bethesda). 2016 Aug 23;6(10):3185–96. doi: 10.1534/g3.116.032508 (PMC5068940; doi:10.1534/g3.116.032508)
Supplement: Supplemental Material [file supp_g3.116.032508_TableS2.pdf]

Table S2 Primers used for sequencing and cloning *cho, ma, mah and red*.

Gene/Primer Use
cho
Cloning Primers:
VhaAC39-1 5' Cloning Primer-ATCGCTCGAGAAGATGAACAGCTCGGGCTT
VhaAC39-1 3' Cloning Primer-CGATTCTAGACGCTTAGAAAATGGGTATGTA
VhaAC39-1 Sequencing Primers
VhaAC39-1 5' UTR-GAACTGCACGCCCTTCGCCA
V1-5-CGGTCGAGCGAGAGCAGTCC
V1-3-CCGAGATGGGTCGCTGGTGC
V2-5-GGAGCCGCTGTCCAACTTTCTGG
V2-3-TGTCGATCTTTGCGCGGTGCTT
V3-5-GGCAACAATCCCGGCGACAAGA
V3-3-CAGAGGCCATCGTTTTAGTCAGAGC
Primers to detect Transposable Element Sites
2176-5n-ACTCTCAGTCGGCGGCTACGTC
XP3 new-GCAGCCAAGCTTTGCGTACTCG
RB5 new-TGACAAGCACGCCTCACGGG
4316-3n-GTGGCGAAGGGCGTGCAGTT
XP-5-TTTACTCCAGTCACAGCTTTG
WH-3-TCCAAGCGGCGACTGAGATG
d02176-5-GCCGTCGCTGGCCGACAATA
d02176-3-TTGCGCGAAAGAGAGGGCGG

Gene/Primer Use

Primers to Sequence CG42541

42-23-AACTCACCGAAATCGAAGGCCC

42-45-ACACAAAGTTATTTTTCAGCGACACCTCT

42-95-ACCCAGCTCGTCGTCAGGCA

42-43-ACGAACTTCGATACCAAACATGAACATTGA

42-75-AGTCTGGAGTTTGGGGACGGGT

42-55-ATGCAGGTATACACCGAGGGCA

42-13-CAAGTCCAGGACGCTGCCACTG

42-85-CCACTTGCACTTCACGTTGGTTGC

42-103-CCTATGCTGCCGCCGCCAAA

42-93-CCTCCGGCAATCCGCCATCG

42-65-CGCTTTGTCAACGGCGCGTA

42-35-CGCTTTTATTGTAATGAAACTTCCGCCGT

42-15-GCAGAGTTGTTGACGCGACATGG

42-73-GCCAGCGGAGGGGATTCTGC

42-25-GCGAGGATCGCTGACCACTCTG

42-105-GCGTCGGATGGTCCGCCAAA

42-63-TCACTGTTCGGAGAAGGCGTCG

42-53-TGTGGAAGCTTGGGATAATCGTGAC

42-83-TGTGTCAGTGGCAGATGGGTGT

42-33-TTCAGCGATACTTACACGACTGCC

42-15n-ACTTGGTTTGCGATGACGACGGA

42-23n-GTGCGGATACTTAATCAAGAAGAAAGAGCA

Gene/Primer Use
42-33n-AAATCGGCGACTTCTGGGCACG
42-3in5-ACACAGGTTAGCAACCAGCAGCC
Primers to Sequence CG42541
42-3in3-ATAGCCACTCTGCTGTTGCACCC
Primers to Sequence CG15239
152-13-AACTGGGCTTCACAGGCAACGG
152-35-AGCGACAACGATACGGATGCGG
152-33-CATGTTGGCAAACTCGCGCTGG
152-25-CGCCAAGCCGGAGAATCCCATC
152-23-GCAGCCGCTGGCTTCTTAACCG
152-13-TGCAAGGGGATTGTATTTTGGGGC
15-15n-CGCAAACGGATTCTGCCTACGCA
15-13n-CAGCTGCGGCGGTACAAGGTATT
maroon
Cloning primers
N8485CL5 - TACCGCGGCCGCCAACATGCCTATCATGTACAACAC
N8485CL3 – ACGGTACCTGACTACTTTCCCTCCAGCATGG
Sequencing primers
8454-15 - ATGAGAAATATCGATGGATTGC

Gene/Primer Use
8454-13 - CGATGCTGTAGGACTCCTTTTC
8454-25 - GACATGGAGGAGCTTATCTGC
8454-23 - GCGATCAGCGTTACGATTTAC
8454-35 - ATCATGAACTCTGACAACAGCG
8454-33 - TACCAAACGGCTGAGTATGACC
8454-45 - ATGCCCATCACCTTCAAACAG
8454-43 - CTGTTGGACTCCCGCATTATC
8454-55 - CTGTCCAATTTGCACGTAAGTC
8454-53 - TCGCATATACCAGACGACCC
8454-65 - ACGATCCTTTTGTTGAGGTCTG
8454-63 - TTATAGTTACTGGCCTCGGCTC
mahogany
Cloning Primers
cl646-5 - TACCGAATTCCGAATGTTCTTCTTCCGTCGCCAGAATG
Cl646-3 – TAACGGTACCTCAGAAGTGCTTGGTCAGATTGCTAAG
Sequencing primers
N646-13 - GGCAGAACCACAACAGCGCC
N646-15 - ACTGGAAGACGAATCTCACGCGG
16-35n - GCATATTGGCCTTCCAGTTTGAC

Gene/Primer Use
6-33n - GAAAGACCCCGATCCTCAGAAGT
6-23n - CCGGTAGTTTGAAGTAGTTCTCGAT
6-25n - CATCCTGTACGTGGTGGATTTG
red Malpighian tubules
Cloning primers
12207CL5-TAAAGCCCCAAACTAAAGTAAAGC
12207CL32-GCTTCTAGACTACAACTCAAAGAAGTCGTCCTG
Exon sequencing primers
12207 1-5-GTATGGCCCCTATGGTGAGTACTTG
red Malpighian tubules Exon sequencing primers
12207B5-ACTTGTTTTTGAGTGCTGCTTG
12207m1-5-CTGGTAACATTGCAGCACGTAG
12207m1-3-CACCTGGGGATAGTACGGTGAA
12207m3-5-CAGGAAATGCATGAACGACTTC
12207m2-3-ATATTTGCGCGACTCAGAAATG
12207m4-5-AAATATGCGCGATACCTTACC
12207m3-3-GGCCAATTATACAGGGCTGACAC
12207m4-3-TGACTTGGCTCGAGATGAGTGC
12207-3U-GCTCTAGACAACATGACTTGGCTCGATATG
12207 1-5-GTATGGCCCCTATGGTGAGTACTTG
12207 1-3-GTGGGACAACAACGAGCAGCTTTTG
12207 2-5-CTCACGCACACACCCACAAAGTG

Gene/Primer Use
12207 2-3-TCGTAATTGGGATTGACACCTACATACAG
12207 3-5-GGCTTAGTCAATGGGAACAGGAGC
12207 3-3-GAGCTTCCCAATAATGTCGTTATGGTGC
12207 4-5-GCGTAATTGATGAGCTCCACCAGC
12207 4-3-CTTCCAGTTTCTATTGGTTCTGTCAGC
12207 5-5-GCTCTAAGGAGTAAGTATTTCCACCAC
12207 5-3-CTGACCAAAGTCATCTCCCCAATG
12207 6-5-GACGCAATCTAGCCTAATTACGCAG
12207 7-5-CGCATCGAACAGATACGGAAGC
12207 6-3-CGAAGCGAGTGGCAGGTTCATTC
R12207f-5-AGCAGAGCAAGAGCCTGGAC
