# Supplementary material for: An Eye on Trafficking Genes: Identification of Four Eye Color Mutations in Drosophila
Source: G3 (Bethesda). 2016 Aug 23;6(10):3185–96. doi: 10.1534/g3.116.032508 (PMC5068940; doi:10.1534/g3.116.032508)
Supplement: Supplemental Material [file supp_g3.116.032508_TableS3.pdf]

Table S3 Sequence differences between the *Drosophila melanogaster* genome sequence and mutant alleles of *maroon, chocolate, and mahogany*.

Position	Change	Exon (E), Intron (I), or UTR (U)	Synonymous (S) or Nonsynonymous
X (R6)		*VhaAC39-1-cho*	
3,882,405	G>T	E	W330L
3R (R6)		*Vps16A-maroon*	
9267133	G>A	E	M4I
9267211	C>T	I	N/A
9267260	G>A	E	A24T
9267444	A>T	I	N/A
9267641	G>A	E	S
9268607-9268615	Deletion	E	Deletion 420-M R I-422
9269662	A>T	E	E712D
9269812	T>C	E	S
9269824	C>G	E	S
9269989	G>A	I	N, N/A
9270091	A>G	E	S
3R (R6)		CG13646-*mahogany*	
24948508	G>T	I	N/A
24949050	C>T	I	N/A
24949116	A>C	E	S

Position	Change	Exon (E), Intron (I), or UTR (U)	Synonymous (S) or Nonsynonymous
24949138	roo insert	E	
24949146	C>G	E*	S
24949176	C>T	E*	S
24949182	C>A	E*	S
24949188	G>C	E*	S
24949334	T>C	E*	I460T
24949455	G>C	E*	S

* These nucleotides are not translated because of the insertion of the *roo* element.
