# Supplementary material for: An Eye on Trafficking Genes: Identification of Four Eye Color Mutations in Drosophila
Source: G3 (Bethesda). 2016 Aug 23;6(10):3185–96. doi: 10.1534/g3.116.032508 (PMC5068940; doi:10.1534/g3.116.032508)
Supplement: Supplemental Material [file supp_g3.116.032508_TableS4.pdf]

**Table S4 Identifiers for proteins used in alignments**

| Identifiers for Putative VhaAC39-1 orthologs (Figure 2) | |
|---|---|
| Species | Identifiers |
| *Drosophila melanogaster* | gi\|17862396\|gb\|AAL39675.1\| LD24653p |
| *Drosophila virilis* | gi\|195399418\|ref\|XP_002058317.1\| GJ16024 |
| *Saccharomyces cerevisiaeYJM1078* | gi\|761519995\|gb\|AJP40571.1\| Vma6p |
| *Aedes aegypti* Mosquito | gi\|157128068\|ref\|XP_001661299.1\| AAEL011025-PA |
| *Apis dorsata* Giant Honey Bee | gi\|572310330\|ref\|XP_006620926.1\| |
| *Tribolium castaneum* | gi\|270006923\|gb\|EFA03371.1\| |
| *Danio rerio* | gi\|82187268\|sp\|Q6PGV1.1\|VA0D1_DANRE |
| *Xiphophorus maculatus* Platyfish | gi\|551530089\|ref\|XP_005816503.1\| |
| *Xenopus laevis* | gi\|27769220\|gb\|AAH42233.1\| |
| *Takifugu rubripes* | gi\|410912365\|ref\|XP_003969660.1\| |
| *Meleagris gallopavo* Turkey | gi\|326927073\|ref\|XP_003209719.1\| |
| *Alligator mississippiensis* | gi\|564262023\|ref\|XP_006269895.1\| |
| *Mus musculus* | gi\|31981304\|ref\|NP_038505.2\| |
| *Homo sapiens* | gi\|19913432\|ref\|NP_004682.2\| |
| *Felis catus* | gi\|410983707\|ref\|XP_003998179.1\| |
| *Ascaris suum* | gi\|541043482\|gb\|ERG82484.1\| |
| *Caenorhabditis brenneri* | gi\|341897984\|gb\|EGT53919.1\| |
| Identifiers for Putative Vps16A orthologs (Fig. 4) | |
| *Drosophila melanogaster* Dmela | Vps16A |
| *Drosophila simulans* Dsimu | GD18603 |
| *Drosophila sechellia* Dsech | GM23792 |
| *Drosophila erecta* Derec | GG16085 |
| *Drosophila yakuba* Dyaku | GE25939 |

| Identifiers for Putative Vps16A orthologs, cont'd. (Fig. 4) | |
|---|---|
| Species | Identifiers |
| *Drosophila ananassae* Danan | GF16588 |
| *Drosophila pseudoobscura pseudoobscura* D pseu | GA21090 |
| *Drosophila persimilis* Dpers | GL13948 |
| *Drosophila willistoni* Dwill | GK22550 |
| *Drosophila virilis* Dviri | GJ10645 |
| *Drosophila mojavensis* Dmoja | GI10429 |
| *Drosophila grimshawi* Dgrim | GH14334 |
| *Musca domestica* | gi|557753230| ref|XP_005176360.1| |
| *Aedes aegypti* | gi|157126533| ref|XP_001660915.1| AAEL010559-PA |
| *Tribolium castaneum* | gi|642940144| ref|XP_008200104.1| |
| *Apis dorsata* | gi|572302945| ref|XP_006617475.1| |
| *Poecilia formosa* | gi|617499503| ref|XP_007540621.1| |
| *Danio rerio* | gi|147899547| ref|NP_001091659.1| |
| *Gallus gallus* | gi|403043595| ref|NP_001258079.1| |
| *Takifugu rubripes* | gi|410906629| ref|XP_003966794.1| |
| *Mus musculus* | gi|19343731| gb|AAH25626.1| |
| *Bos taurus* | gi|62751411| ref|NP_001015522.1| |
| *Anolis carolinensis* | gi|637367371| ref|XP_008121385.1| |
| *Felis catus* | gi|755708308| ref|XP_011279227.1| |
| *Macaca fascicularis* | gi|355784645| gb|EHH65496.1| |
| *Caenorhabditis elegans* | gi|17552018| ref|NP_498411.1| VPS-16 |
| *Ascaris suum gi|541048773|* | gi|541048773| gb|ERG87472.1| |
| *Saccharomyces cerevisiae P283* | gi|584473684| gb|EWH15448.1| Vps16p |
| *Alligator mississippiensis* | gi|564267015| ref|XP_006272323.1| |
| *Xenopus (Silurana) tropicalis* | gi|52345856| ref|NP_001004976.1| |
| *Homo sapiens* | gi|17978479| ref|NP_072097.2| |
| *Rattus norvegicus* | gi|53850610| ref|NP_001005541.1| |

| Identifiers for Putative Drosophila CG13646 orthologs (Genes) (Fig. 6) | |
|---|---|
| *Drosophila melanogaster* | G13646 |
| *Drosophila simulans* | GD21153 |

| Identifiers for Putative Drosophila CG13646 orthologs (Genes) cont'd. (Fig. 6) | |
|---|---|
| *Species* | Identifier |
| *Drosophila sechellia* | GM26650 |
| *Drosophila erecta* | GG11342 |
| *Drosophila yakuba* | GE23537 |
| *Drosophila ananassae* | GF16317 |
| *Drosophila pseudoobscura pseudoobscura* | GA12432 |
| *Drosophila persimilis* | GL24405 |
| *Drosophila willistoni* | GK12978 |
| *Drosophila virilis* | GJ24341 |
| *Drosophila mojavensis* | GI22220 |
| *Drosophila grimshawi* | GH19450 |

| Identifiers for Putative Drosophila CG12207 Lys M orthologs (Fig. 8) | |
|---|---|
| Species | Identifiers |
| Dmel = *D. melanogaster* | CG12207 |
| Dwil = *D. willistoni* | GK11342-PA |
| Dgri = *D. grimshawi* | GH18663-PA |
| Dvir = *D. virilis* | GJ14410-PA |
| Dmoj = *D. mojavensis* | GI10811-PA |
| Dpse = *D. pseudoobscura* | GA11477-PA |
| Dper = *D. persimilis* | GL23268-PA |
| Dana = D. *ananassae* | GF16161-PA |
| Dere = *D. erecta* | GG16845-PA |
| Dyak = *D. yakuba* | GE24226-PA |
| Dsec = *D. sechellia* | GM24156-PA |

| Identifiers for Putative Drosophila CG12207 Lys M orthologs (Fig. 9) | |
|---|---|
| Species | Identifiers |
| Alligator-*Alligator mississippiensis* | gi\|564233535\|ref\|XP_006261132.1\| |
| *Arabidopsis-Arabidopsis thaliana* | gi\|30682281\|ref\|NP_196437.2\| |
| Bee-*Apis dorsata* | gi\|572271887\|ref\|XP_006613869.1\| |

| Identifiers for Putative Drosophila CG12207 Lys M orthologs, cont'd (Fig. 9) | |
|---|---|
| Chicken-*Gallus gallus* | gi\|513201397\|ref\|XP_004943854.1\| |
| Ciona-*Ciona intestinalis* | gi\|198433694\|ref\|XP_002130231.1\| |
| Collared flycatcher-*Ficedula albicollis* | gi\|525008984\|ref\|XP_005052132.1\| |
| D melanogaster-*Drosophila melanogaster* | gi\|24646795\|ref\|NP_731895.1\| |
| D virilis-*Drosophila virilis* | gi\|195399652\|ref\|XP_002058433.1\| |
| Flour Beetle-*Tribolium castaneum* | gi\|189239121\|ref\|XP_001815551.1\| |
| *Xenopus-Xenopus laevis* | gi\|308153293\|ref\|NP_001184014.1\| |
| Takifugu-*Takifugu rubripes* | gi\|410912437\|ref\|XP_003969696.1\| |
| Golden-collared manakin- *Manacus vitellinus* | ACC KFW83668.1 gi\|679193756\| |
| House cat-*Felis catus* | gi\|410968324\|ref\|XP_003990657.1\| |
| House fly-*Musca domestica* | gi\|557768669\|ref\|XP_005184011.1\| |
| *Homo sapiens*-Human | gi\|159164152\|pdb\|2DJP\|A |
| Corn-*Zea mays* | gi\|413916929\|gb\|AFW56861.1\| |
| Malo sina-*Oryza brachyantha* | gi\|573948441\|ref\|XP_006656525.1\| |
| Mosquito-*Aedes aegypti* | gi\|157167545\|ref\|XP_001654849.1\| |
| Mouse-*Mus musculus* | gi\|130490961\|ref\|NP_081585.2\| |
| Opossum-*Monodelphis domestica* | gi\|126313718\|ref\|XP_001366372.1\| |
| Rainbow smelt2 -*Osmerus mordax* | gi\|225708028\|gb\|ACO09860.1\| |
| Sea Urchin-*Strongylocentrotus purpuratus* | gi\|390348158\|ref\|XP_786139.2\| |
| Slime mold-*Polysphondylium pallidum PN500* | gi\|281201963\|gb\|EFA76170.1\| |

| Identifiers for CG13646 putative protein orthologs in mammals and insects (Fig. S1) | |
|---|---|
| Species | Identifiers |
| Part A | |
| *D. melanogasster* | gi\|24649865\|ref\|NP_651316.1\|G13646 |
| *D. simulans* | gi\|195573673\|ref\|XP_002104816.1\| |

| Identifiers for CG13646 putative protein orthologs in mammals and insects, cont’d (Fig. S1) | |
|---|---|
| Species | Identifiers |
| *D. pseudoobscura pseudoobscura* | gi\|198451685\|ref\|XP_001358480.2\| |
| *D. virilis* | gi\|194152331\|gb\|EDW67765.1\| |
| *Aedes aegypti* | gi\|157124582\|ref\|XP_001654116.1\| |
| Flour Beetle-*Tribolium castaneum* | gi\|642939900\|ref\|XP_0082002 |
| Silkworm-*Bombyx mori* | gi\|512910840\|ref\|XP_004927269.1 |
| Parasitic wasp-*Nasonia vitripennis* | gi\|156549334\|ref\|XP_001601210.1\|_1 |
| Pea aphid- *Acyrthosiphon pisum* | gi\|641671950\|ref\|XP_008185768.1\| |
| Human body louse- *Pediculus humanus corporis* | gi\|212505775\|gb\|EEB10155.1\| |
| Human body louse- *Pediculus humanus corporis* | gi\|242003872\|ref\|XP_002422893.1\| |
| Part B | |
| *D melanogaster* | gi\|7303217\|gb\|AAF58280.1\| |
| *Mus musculus* | gi\|125490380\|ref\|NP_033534.2\| |
| *Homo_sapien* | gi\|17999520\|ref\|NP_542119.1\| |
| *D. melanogaster* | gi\|24649865\|ref\|NP_651316.1\|G13646 |
