# Supplementary material for: An Eye on Trafficking Genes: Identification of Four Eye Color Mutations in Drosophila
Source: G3 (Bethesda). 2016 Aug 23;6(10):3185–96. doi: 10.1534/g3.116.032508 (PMC5068940; doi:10.1534/g3.116.032508)
Supplement: Supplemental Material [file supp_g3.116.032508_TableS5.pdf]

Table S5 Distribution of CG122207 and CG3259 substitutions in *red*[1], *red*[K1] and OreR stocks.

CG12207					CG3259				
Strain	Number and type of nucleotide changes[a]				Strain	Number and type of nucleotide changes[a]			
		M	S	U			M	S	U
red[1] only	11	1	0	10	*red*[1] only	11	4	5	2
red[1] and *red*[K1]	1	0	1	0	*red*[1] and *red*[K1]	1	0	1	0
red[1] and OreR	5[b]	0	3[b]	2[b]	*red*[1] and OreR	0	0	0	0
red[K1] only	3	1	1	1	*red*[K1] only	1	0	1	0
red[K1] and OreR	5	0	0	5	*red*[K1] and OreR	4	0	1	3
OreR only	0	0	0	0	OreR only	0	0	0	0
red[1], *red*[K1] &OreR	9	0	0	9	*red*[1], *red*[K1] &OreR	8	5	3	0
Total *red*[1]	26				Total *red*[1]	20			
Total *red*[K1]	18				Total *red*[K1]	14			
Total OreR	14[c]				Total OreR	12			
Total No. Sites		**2**	**5**	**27**	Total No. Sites		**9**	**11**	**5**

a. M is missense mutation; S is synonymous mutation; U is untranslated mutation.

b. At least one OreR individual was heterozygous for the Genbank and *red*[1] nucleotides.

C, The OreR heterozygotes were not counted as having substitutions.
